# Towards a theory of morphosyntactic focus marking

**DOI:** 10.1007/s11049-023-09567-4

**Published:** 2023-01-27

**Authors:** Muriel Assmann, Daniel Büring, Izabela Jordanoska, Max Prüller

**Affiliations:** 1 Institute of Linguistics, University of Vienna, Sensengasse 3a, 1090 Vienna, Austria; 2 CNRS-LACITO, 7 rue Guy Môquet (bât. D), 94801 Villejuif Cedex, France

**Keywords:** Focus, Morphological focus marking, Focus alternatives, Blocking, Focus ambiguity, Focus in African languages, Unalternative semantics

## Abstract

Based on six detailed case studies of languages in which focus is marked morphosyntactically, we propose a novel formal theory of focus marking, which can capture these as well as the familiar English-type prosodic focus marking. Special attention is paid to the patterns of focus syncretism, that is, when different size and/or location of focus are indistinguishably realized by the same form.

The key ingredients to our approach are that complex constituents (not just words) may be directly focally marked, and that the choice of focal marking is governed by blocking.

## Introduction

Languages in which focus is marked by special morphemes or syntactic positions (henceforth MorFoc languages) have long been known and also, more recently, well described. Yet research on focus in MorFoc languages and theoretical research on focus in English (and other Germanic languages) have been without significant points of contact. Technical concepts used in the discussion of English focus, such as Projection, Prosodic Defaults, or AvoidF, play little or no role in research in MorFoc languages. Conversely, few, if any, attempts to refine the systems proposed for English focus based on empirical findings in MorFoc languages have been made. A comprehensive cross-linguistic *theory* of focus marking has not been pursued.

This paper attempts to fill this gap. Based on a survey of over 20 MorFoc languages, we found two ingredients to be crucial in analyzing the focus marking patterns found there:

Direct Marking/No Projection: Each focal marking marks exactly one constituent as focal; crucially, this constituent may be non-terminal and in fact as big as an entire clause.

Blocking: One has to choose the most specific focus marker that is pragmatically appropriate.

Some form of Blocking is assumed in many theories of focus in English.^1^ But in these theories, focal marking is usually anchored on words or morphemes, and may then “project” to non-terminals, potentially yielding focus syncretisms, i.e. one prosodic pattern marking different sizes of focus in different contexts (we prefer “syncretism” over the more common term “focus ambiguity” because we will argue that there is no structural distinction at all between different focus sizes marked by the same marking). For example, *Mary ate YOGURT* with the main pitch accent (indicated by capitals) on *yogurt*, may mark as focused either just the accented word, or bigger constituents containing it—here: VP and S—and hence can be used in any context pragmatically requiring either one of these focus sizes. Projection theories model this by having focus projection rules set up in such a way that the focus can “project” from the object to the VP to the clause, but not, say, to the subject or the verb alone (see, e.g., Selkirk 1984, 1995; Rochemont 1986; Schwarzschild 1999 among many others). It is immaterial in this connection whether the projection rules actually work from words to potentially focal phrases (“bottom-up”) or from the focal phrase to the word bearing the nuclear accent (“top-down”) (as, e.g., in von Stechow and Uhmann 1986 or Uhmann 1991: Ch. 5); what is crucial is that the rules syntactically define the set of possible pairings of a prominent word and the different constituents in a tree containing that word that can be marked as focal by it.

In the present paper, as opposed to that, we argue that in MorFoc languages, each focal marking (e.g. morpheme and/or position) marks one and the same particular constitutent (say, the object) in all structures and contexts it occurs in; that constituent, in turn, need not be a terminal (word or morpheme), but may be a phrase or even a whole clause. This does not entail that there are no focus syncretisms in MorFoc languages. There are. But rather than assuming that focus is marked on constituent A, and then may “project” to constituent B, then C…and possibly ultimately on to yet a bigger constituent D, on our account, the marking goes to D (i.e. the biggest constituent A could, in a projection theory, project to). Focus on D, however, may potentially be used in cases the context requires focus on D, *or any sub-constituent of D*, including C, B and A.

This may sound like a different way of saying the same thing projection theories say, but it is not, once Blocking is included in the picture. Due to Blocking, morphosyntactically marking D (the big constituent) may only be used to mark pragmatic focus on, say, B (the sub-constituent) if there isn’t a different morphosyntactic marking for B alone. So whether or not a marking is syncretic, and, in case it is, which focus sizes it can mark, depends, in the account proposed here, on the inventory of other focus markings in the language, and nothing else. On projection accounts, it depends on the specific focus projection rules.

One central result of our survey is that the patterns of syncretism vary vastly from language to language (as we discuss in detail in Sects. 2, 3 and 5). For example, seven out of 21 languages mark clausal focus and *subject* focus in transitive sentences in the same way, rather than clausal focus and focus on (a part of) VP, as is the case in Germanic and Slavic languages, as well as in Italian and Spanish. VP focus in turn is syncretic with object focus (like in the European languages mentioned) in three MorFoc languages in our sample, but syncretic with V focus in six (and with both in another two).

Once we identified for each morphosyntactic marking the constituent it marks as focal, the Blocking principle correctly describes all of these different patterns of syncretism on the basis of each language’s inventory of focus markings alone. A projection account, as opposed to that, would presumably have to adjust the focus projection rules language by language, missing the fundamental parallelisms expressed by Blocking. And even one of the most basic predictions of any projection theory, that every syncretism includes a word level focus (namely the word from which the projection starts) does not hold universally: our sample includes at least three cases in which clausal focal marking is not syncretic to *either* subject or VP focus (or any other constituent focus), arguing strongly that the very idea of projection from (or “percolation” down to) a word is not cross-linguistically applicable (Sect. 4, especially 4.2.2).

An existing alternative to syntactic focus projection theories are theories in which the exponent of the focus (in the case of English: the word bearing the nuclear pitch accent) is determined by *prosodic* defaults within the constituent that is the pragmatic focus (e.g. Jackendoff 1972; Truckenbrodt 1995; Zubizarreta 1998; Reinhart 2006). What is attractive about such theories is that they replace specific, syntactic focus projection rules by independently motivated prosodic rules, such as nuclear accent placement or prosodic phrase formation. It remains obscure, however, how such theories could generalize to MorFoc languages: on the one hand, prosodic properties play little, if any, identifiable role in focal marking in MorFoc languages; on the other, we are not aware of, nor have we been able to discern in our data, a comparable independently motivated notion of “default morpheme placement within a syntactically complex constituent.”^2^ Furthermore, in more than a few cases (involving at least eight languages in our sample) the pragmatically focused constituents does not even *contain* the focus marking morpheme (some containing constituent does), contradicting the very notion of “finding the exponent within the focus” (Sect. 4.2.1).

Does our proposal imply, then, that MorFoc languages require an entirely different kind of focus theory than, say, English? We do not think so at all. As we show in Sect. 6.4, an analysis of English focusing based on prosodic defaults is readily formulated within the exact same framework we use in our analysis of MorFoc languages: a smaller focus is syncretic with a larger one if and only if there is no specific focal marking for the smaller one. We pursue the idea that English marks focus by reversing prosodic defaults between sister nodes, while prosodic default structures are neutral with regard to focusing (an idea traceable, once again, to Williams 1997, at least). The rest is again due to Blocking: default prosody within a constituent C may mark any pragmatic focus that cannot be marked by reversing the default some place within C. The difference between the MorFoc languages on the one hand, and a prosodically marking language like English on the other, lies solely in the fact that English has the means of focal marking at every branching node (though not for every daughter of a branching node, Sect. 4.4), including all the way down to the terminals, whereas MorFoc languages don’t (they are generally restricted to one focal marking per clause). Everything else is the same.

Still, in order to introduce and motivate our proposal, we will start with the unfamliar—MorFoc languages—postponing comparison with existing systems until Sect. 4.

## Introducing the proposal

### Case study I: Gùrùntùm

As a first illustration, consider the case of Gùrùntùm (also known as Gùrdùŋ), a South Bauchi (West Chadic, Afro-Asiatic) SVO language spoken in Bauchi State in Nigeria by 15,000 people (1993; Eberhard et al. 2022), as described in Hartmann and Zimmermann (2009) (henceforth H&Z 2009). Gùrùntùm employs a focus marking morpheme , which may occur in three basic configurations. When preceding the subject, it marks subject focus, as in (1).^3^ The pragmatic focus is indicated by underlining in the translations throughout.

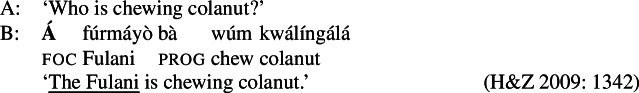

When  occurs between the verb and its following argument, as in (2), the sentence can express object focus, narrow verb focus or VP focus. Thus (2) could answer any of the questions: ‘What is he gathering?’ ‘What is he doing with the seeds?’ and ‘What is he doing?’^4^ (The three underlines in (2) each correspond to a different pragmatic focus, i.e. object focus, narrow verb focus or VP focus.)

(2)
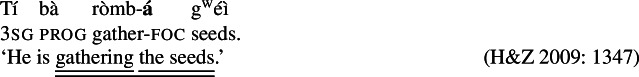

Lastly,  at the end of a clause marks clausal focus. The example in ((3)), according to Hartmann and Zimmermann (2009), is used discourse-initially:

(3)
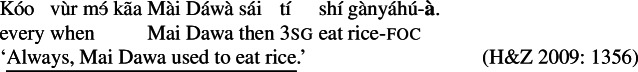

We say that each of (1)B, (2) and (3) instantiates a distinct Focal Marking; depending on the language, focal markings may differ from one another in the placement of focus marking morphemes (such as in Gùrùntùm (1)–(3)), but also by including different lexical focus marking morphemes, different constituent orders, or, as is familiar from European languages, different intonations.

In contradistinction to the form-related “focal marking,” we use the terms “subject focus,” “object focus,” “VP focus,” etc. in a *pragmatic* sense. A sentence is said to express (or simply “have”) X focus (as marked by underlining in the preceding examples and throughout) if it can felicitously be used to correct another sentence S′ which differs from S only in that all of X is replaced by something different in S′, or if it can felicitously be used to answer a question Q whose wh-element corresponds to X in S; these are the standard diagnostics for “being the focus,” but exclude so-called *verum* or *polarity* focus, which we do not explore in the present study, because its is not clear that verum marking is, cross-linguistically, related to focusing (see Romero and Han 2004; Zimmermann and Hole 2008; Schwarz 2010; Gutzmann and Castroviejo Miró 2011; Matthewson 2017; Goodhue 2018; Gutzmann et al. 2020; Matthewson 2020 for discussion). Where the same sentence/focal marking can express different foci, we speak of focus syncretism (see Sect. 2.2 for why we eschew the more familiar term “focus ambiguity”).

Before we proceed to present our proposal, two disclaimers are in order. First, we do not attempt to model the precise contributions that each indivdual focus marking morpheme makes, as we are interested in the properties of the focal markings systems as a whole, not their particular morphological, syntactic or phonological realization. For this reason, too, we use the gloss foc for any morpheme that distinguishes some focal marking from another, irrespective of whether that morpheme is, on final analysis, a dedicated focus marking morpheme, or has other functions as well.

Second, the focal markings we discuss are not obligatory in every instance of focusing in the languages considered in the present paper. For example, it is sometimes possible to express sentence focus in Gùrùntùm without the sentence final ; as Hartmann and Zimmermann (2009: 1359–1360) discuss, the occurrence (or not) of  in sentence focus is regulated by other grammatical and discourse factors, e.g. the verbal aspect (it only appears in perfective and presentational sentences), or the structuring of information blocks in a larger discourse (see Hartmann and Zimmermann 2009: Sect. 6, for a general discussion of these factors). We do not believe nevertheless that this affects our proposal, as we are interested in what the focus marking morphemes do when present, and which types of syncretisms occur in general.

### Basic focal marking: No projection

Our analysis starts by postulating for each focal marking exactly one constituent that is thereby focally marked. This is illustrated for Gùrùntùm in (4); for perspicuity we color the focally marked constituent and the morpheme marking it in the trees (color figures are available in online version of paper).

(4)
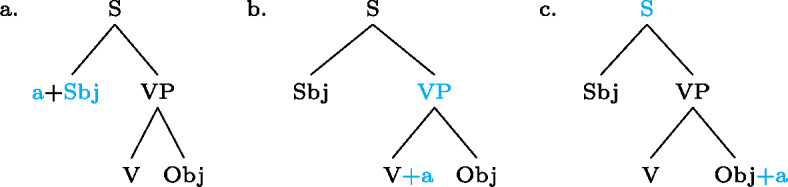

Semantically, focally marking a constituent plays a very similar role to assigning an [F]-marker in theories like Rooth (1992): the focus alternatives of a focally marked constituent are meanings of the same semantic category (type); constituents “outside” the focally marked ones do not introduce alternatives (just like [F]-less nodes in Rooth’s approach); from this it follows that a focal marking can never realize a focus that is bigger than the focally marked constituent.

We can think of the focal markings in (4) as focally marking the S, VP and Sbj nodes, respectively, in the same way a pitch accent licenses [F]-marking on a (pre)terminal in Selkirk’s (1984) Basic Focus Rule. We will discuss the relation between the placement of the focus marking morpheme and the constituent it focally marks in Sect. 4 below; for now we just stipulate the markings.

(4a) is a rather straightforward case: the subject DP is focally marked, so this is the form to use when one wants to focus the Sbj, i.e. needs non-trivial alternatives to the subject meaning. In (4b)  is taken to directly focally mark VP (rather than V or Obj). But this does not translate into “(4b) is VP focus.” Rather, it translates into “(4b) may be used if VP *or something within it* is the focus.”^5^ So in fact, (4b) is syncretic for V, VP and Obj focus. This is a significant departure from the usual way of thinking about focus syncretism: rather than saying that the same focal marking, say , is structurally ambiguous between V-, Obj- or VP-focus, we take it to unambiguously focally mark VP (the focus size that encompasses all others), which is semantically general enough to allow for all V-only and object-only alternatives. This is why the distinction between the (pragmatic) focus and the focally marked constituent is important: according to our analysis, they do not always coincide. This will be discussed in detail in Sect. 4.4 below. Focally marking VP directly in (4b) illustrates what was meant by “No Projection” earlier: broad foci do not project from narrow foci by specialized projection rules, they are directly licensed by morphological focal marking.

In (4c), finally, the root node is focally marked. This means this structure can be used to realize clausal focus. Considering what we just said about (4b), it in fact means that it can be used to mark S *or anything within S* as focus. But as a matter of fact, (4c) can *only* realize clausal focus; it is not syncretic with any other focus size. This is captured by the second ingredient of our analysis, Blocking.

### Blocking

Our proposal is that focally marking the clause in Gùrùntùm in (3) or ((5)) cannot be used to express Sbj or VP focus (or any other focus smaller than those) precisely *because* Gùrùntùm has specialized focal markings to realize Sbj focus and VP-focus (and hence any foci *within* those constituents as well).

(5)
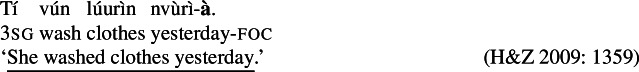

This is the Blocking effect. Crucially, this effect hinges on the inventory of focal markings the language has. For example, Gùrùntùm does not have specialized markers for focally marking V or the XP following it within VP; consequently, (2)/(4b) can also be used to realize V or XP focus, i.e. the focal marking is syncretic, unlike (5)/(4c). But, to reiterate, the fact that focally marking the clause in Gùrùntùm can*not* mark any sub-clausal focus has nothing to do with the way this marking comes about (like focus projection rules), but only, via Blocking, with what other focal marking possibilities the language has.

## Three further case studies

Before spelling out more details of our analysis, let us briefly illustrate its general workings with three further case studies, namely Buli, Hausa and Wolof. Unless otherwise specified, the data are obtained from elicitation, conducted by e-mail, video call and/or in person with a total of 13 consultants, six for Hausa, six for Wolof and one for Buli. Aside from translations and felicity judgments with contexts, we also used visual stimuli, which were partly taken from the questionnaire developed by Skopeteas et al. (2006) and partly self-made. Tone is transcribed where the consultants indicated it.

### Buli

Buli is a Mabia/Gur language of the Niger-Congo family, spoken by 168.000 speakers in northern Ghana (Eberhard et al. 2022). The canonical word order is SVO. It has three distinct focus marking patterns: a morpheme ,^6^ following the subject (optionally combined with  preceding it), which is used for subject or clausal focus, ((6)); a morpheme  which precedes the direct object and which marks VP or object focus, ((7)); and a morpheme , following the VP, which marks narrow V focus, (8).^7^

(6)
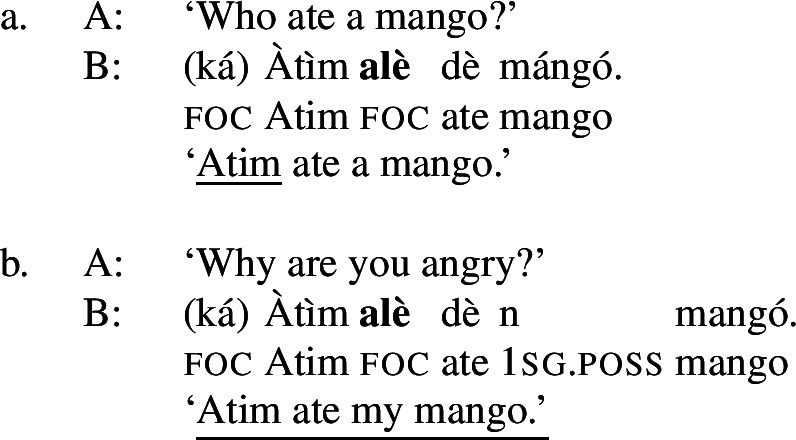

(7)
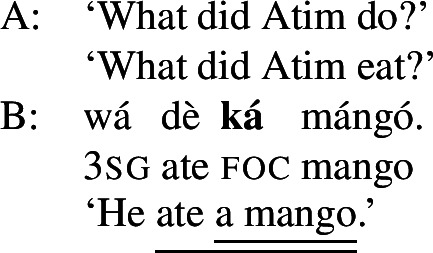

(8)
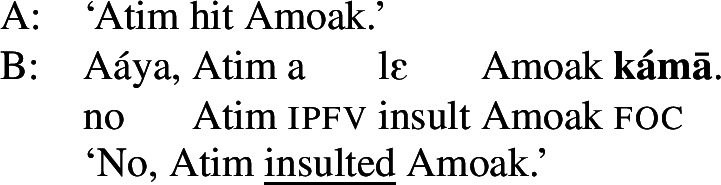

As with Gùrùntùm, we start by assigning to each of those markings exactly one constituent thereby focally marked:

(9)
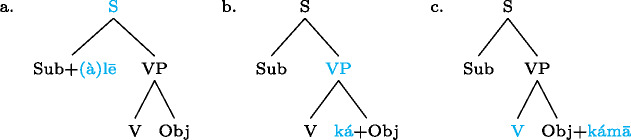

Following the logic of Blocking introduced above,  in (9a) can be used where S or any sub-constituent thereof are the focus, except those for which there are more specialized markings.^8^ Since Buli has a specialized way for focally marking VP, (9b), this in effect restricts (9a) to S focus and Sbj focus. By the same reasoning,  in (9b), which focally marks VP, could be useable as VP-, V- or Obj focus; but since there is a specialized V focal marking, (9c), only VP- and Object focus are in fact realized by .

Note that in this way we derive the—from a European point of view—unusual pattern of focus syncretism found in Buli: clausal focus and Sbj focus are realized in the same way.^9^ We also derive the more familiar looking syncretism between Obj- and VP focus.^10^

In traditional parlance, one would say that in Buli, subject focus “projects” to the clausal node. On the present analysis, it is more accurate to say that, because Buli does not have a dedicated subject focal marking, subject focus is realized by focally marking the clause; the underlying logic of the Sbj/clausal focus syncretism is the same as in other, more familiar looking syncretisms such as VP/Obj. This is where our proposal differs from the otherwise similar one in Schwarz (2016), who treats Sbj and clausal focus as *pragmatically* identical, generally calling sentences with  “thetic,”^11^ Given that  clearly appears in categorical contexts such as (6a), Schwarz (2016) effectively claims that a sentence with  is formally marked as thetic, i.e. as having no internal information structure, while at the same time being interpreted as Sbj focus, a prototypical kind of categorical sentence. Our proposal simply analyzes  as focally marking the clause, but useable to express either Sbj or clausal focus.

### Hausa

Hausa (West Chadic, Afro-Asiatic) has two different focus strategies: In-situ focus and ex-situ focus. We will discuss the ex-situ strategy in detail in Sect. 7, and restrict the discussion to canonical order examples here.

Hausa makes a two-way formal distinction, between (part of) subject focus on the one hand, and every other focus, including clausal focus, on the other. (10) is an example of subject focus, taken from Hartmann and Zimmermann (2007c) (henceforth H&Z 2007c).

(10)
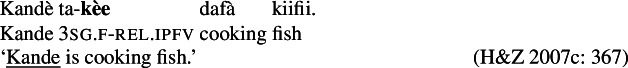

The focus in (10) is marked by the so-called relative form  on the pre-verbal Person-Aspect-Complex (PAC); see Tuller (1986). Unless the relative form is impossible for independent reasons (for example, the relative marking never occurs in future, habitual, subjunctive and negative clauses; see Hartmann and Zimmermann 2007c: 368; Newman 2000; Jaggar 2001) its use is obligatory in subject focus sentences.^12^

Foci other than narrow Sbj are expressed using the absolute form of the PAC, such as  in ((11)) (alternatively, non-subject foci may be marked by movement, as discussed in Sect. 7). An answer like (11)B can be used in all-new contexts, as well as answering any constituent question, as long as it is not asking about the subject:^13^

(11)
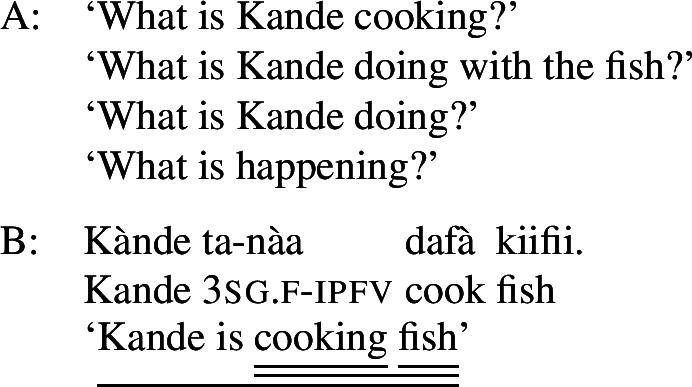

Following Hartmann and Zimmermann (2007c), we consider the absolute form as the default, i.e., as the absence of a specific marking.

In addition to the relative form of the PAC, Hausa has the morpheme , which is plausibly analyzed as a focus marker (Green and Jaggar 2003; Green 2007). It can optionally occur after the focus, as shown in ((12)), where it follows the subject. It furthermore agrees in gender with the preceding noun— for feminine,  for other, and has polar tone, i.e., a tone that is the opposite of the preceding one (Parsons 1963).

(12)
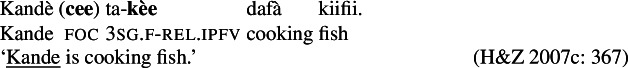

The morpheme  can also be used with non-subject focus. In this case, it is often placed sentence-finally, rather than directly following the pragmatic focus. In our own data, we only have sentence-final instances of the morpheme. However, there is another possible in-situ position for it: after the object in an SVOX sentence, in which case it can be followed by an adjunct (Newman 2000; Hartmann and Zimmermann 2007a). We return to this issue in the discussion at end of this section, for now we only show instances of sentence-final , such as ((13)).

(13)
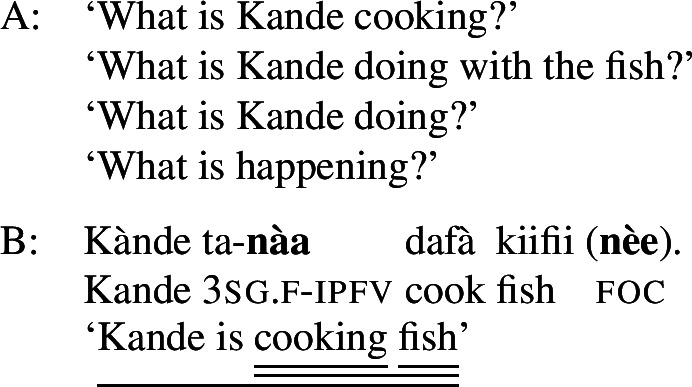

So, even in the case where ((13)) is an answer to the question ‘What is Kande doing with the fish?’—i.e. verb focus— still appears clause-finally. Note, too, that in (13)  appears in its default form  clause-finally—even if it immediately follows a feminine, pragmatically focused noun, such as *àyàbà* ‘banana,’ as in ((14)).

(14)
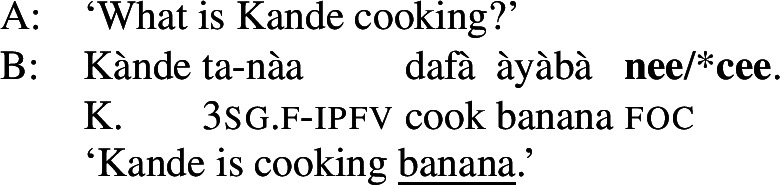

As Green and Jaggar (2003: 198) point out, placement and non-agreement together strongly imply that structurally, sentence-final  is indicative of clausal focus, even where pragmatic focus is on a sub-constituent. In the same vein, Hartmann and Zimmermann (2007c: Sect. 5) show that there is no prosodic difference depending on what the pragmatic focus is in sentences like (13) either. Thus, S, VP, V and Obj focus are syncretic in Hausa. The subject-initial structure with the relative form (with or without ), on the other hand is syncretic merely between subject focus, as in (10) and (12) above, and part-of-subject focus, as in (15).

(15)
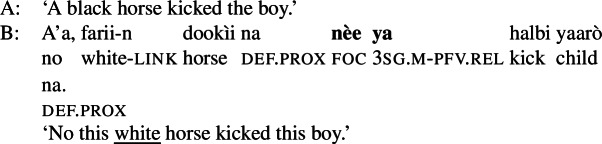

We analyze the relative form (and optional post-subject ) to focally mark the Sbj, and its absence (plus optional clause-final ) to focally mark the clause, see (16).

(16)
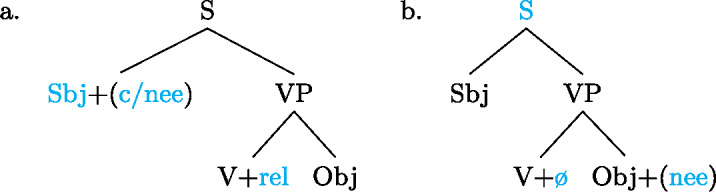

The absolute form of the verb in (16b) focally marks the S node, so it may express clausal focus, but also any other focus, provided it is not (within) the subject: since the language has a dedicated Sbj focal marking available, (16a), Blocking prevents the clausal focal marking from expressing those.

Returning to the issue of the position of in-situ : since  *can* attach to the object in an SVOX clause, as in ((17)), one could postulate that in this specific case it functions as a local marker.

(17)
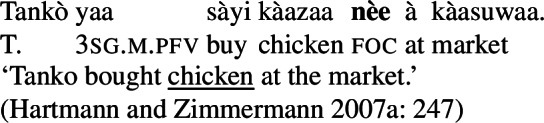

However, if  were a local marker in ((17)), one would expect it to agree in gender with the feminine noun  ‘chicken’ and show up as . An alternative analysis would be that, since according to Newman (2000) the Hausa core clause consists of [S TAM V OBJ], everything that follows the object is right-attached, i.e., outside of the core clause. In this case, ((17)) is simply another example of “sentence-final” . We leave the specifics of this issue for further research.

### Wolof

Wolof is an Atlantic (Niger-Congo) language spoken predominately in Senegal and the Gambia by approximately 10 million people (Eberhard et al. 2022). It has SVO(X) word order. Focus in Wolof is marked on what Robert (1991) has termed the “verbal conjugation” which occurs pre- or post-verbally and encodes subject person and number, aspect and mood.

This verbal conjugation marker changes depending on whether the focus is a subject, as in (18a), non-subject, as in (18b), or verb/VP, as in (18c) (Robert 1991). We will refer to the subject focus conjugation as the -form, the verb/VP focus conjugation as the -form and the object focus conjugation as the -form.^14^

(18)
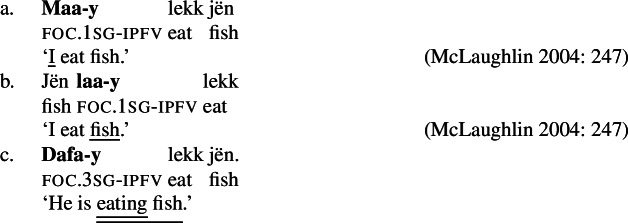

Verb and transitive VP focus are syncretic in Wolof, i.e., (18c) can answer both the questions “What is Omar doing?” and “Is he buying fish?” This is another syncretism we don’t find in, for example, Germanic languages.^15^

All of the examples in ((18)) are in the imperfective aspect, marked by the *y*-suffix. Unlike the term focus marking morphemes, the clausal focus marking morpheme varies depending on which aspect it occurs with. Clausal focus with the progressive aspect is marked with a -form, as in ((19)). The -form is refered to as “sentence focus” in Ngom (2003), but more commonly as “progressive” (Torrence 2013) or “presentative” (Robert 1991).

(19)
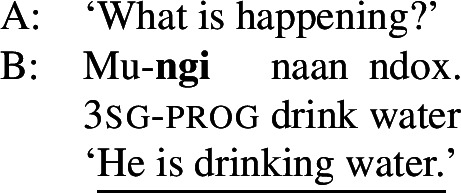

Clausal focus with the perfective aspect is marked with a -form, refered to as “sentence focus” in Russell (2006), as in ((20)).^16^

(20)
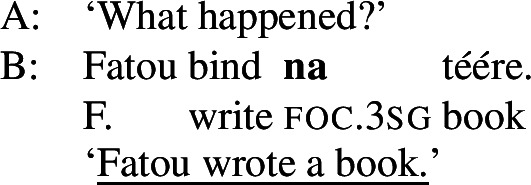

We henceforth only illustrate clausal focus in the perfective aspect.

As can be seen in (18b), object focus in Wolof is not just indicated by the verbal conjugation, but also by movement of the object to a clause-initial position. In fact, according to Martinović (2013),  is only a reflex of movement, which is the actual marker of focus. Focus movement will be discussed in Sect. 7. For now, leaving movement aside, the trees showing the syncretisms in Wolof look as in (21):

(21)
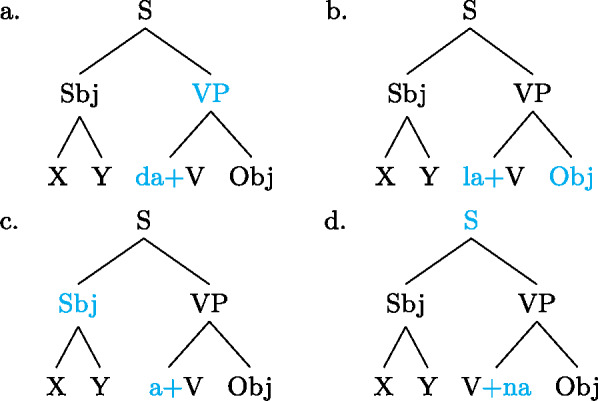

We analyze  as marking the VP as focal, (21a), while the construction with  marks the object as focal, (21b). By Blocking, this prevents focal marking on the VP from expressing object focus. However, since there is no focal marking that specifically marks the *verb* as focal, VP focus and verb focus are syncretic in Wolof.  focally marks the Sbj, (21c).  marks the entire clause as focal, and, since every other node has a more specific marker, it can only be used for clausal focus, (21d). For more details on the Wolof focus marking system see Njie (1982); Robert (1989, 2010); Ngom (2003); Torrence (2013); Martinović (2015).

These case studies conclude the intial presentation of our proposal. Summarizing, our theory designates, for each focal marking, one node thereby focally marked. The designated node thus sets the maximal size of focus that can be realized by the marking in question. In principle, any node dominated by (“included in”) the designated node could also be “the focus,” subject to Blocking. The minimal size of a focus is thus systematically determined by the maximal size of other focal markings in the language.

Note that the latter concept, the “minimal size” of focusing indicated by a given marking, is alien to familiar focus theories, as the minimal size of focus for *any* marking in European languages appears to be the syllable carrying the pitch accent. But this is evidently not the case e.g. in Gùrùntùm, where the minimal size of focus realized by a clause final  is the entire clause.

## Comparison with existing accounts

### Existing approaches

Most descriptive works on morphological focus-marking languages (MorFoc languages), such as the ones we quote in this paper, are cast in terms of “the focus in sentence S is on constituent X, and is realized by…” (see e.g. the survey in Kalinowski 2015). The analysis presented in the previous sections started from basically that perspective, adding a number of theoretical refinements, in particular: how to derive patterns of syncretism (answer: focally marking vs. pragmatic focus), and, at the same time, how to predict when a narrow, rather than a broader focal marking will realize a particular focus (answer: Blocking).

Theoretical questions like these are of course at the heart of various accounts of focusing in English. We believe that, once we adjust such theories to the specific challenges posed by MorFoc languages, the proposal outlined in the previous sections is in fact a quite conservative adaption of them—despite its radically different appearance. We will now trace the way from existing theories to the present proposal; for reasons of space and generality, we will proceed from a high-level perspective. As our stand-in for any number of theories for English, we use (22).

(22) Focus Theory E(nglish):
  Any constituent that contains the word bearing the nuclear pitch accent and displays “default prosody” internally may be the focus of a sentence.

(22) transparently and accurately describes a long line of theories starting with Jackendoff (1972), and including, with various variations on the theme, Truckenbrodt (1995); Zubizarreta (1998); Reinhart (2006); among others; all of those take the “default prosody” mentioned in (22) to be exclusively determined by morphosyntactic factors such as linear order, syntactic category, or embedding. A second sub-group of approaches, which we distinguish from the ones just mentioned where necessary, take default prosody to be itself a matter of focus marking, subject both to specific projection rules and pragmatic conditions, e.g. Selkirk (1984, 1995); Rochemont (1986); Schwarzschild (1999); as well as Gussenhoven (1983). Though these latter approaches typically do not make reference to the nuclear pitch accent in their focus rules, they all hold that only focus-marked terminals can bear pitch accents, which entails that the *nuclear* pitch accent is part of a focus; furthermore, for any given context (in particular: any determination of which elements are given and which are not) their rules uniquely determine one and only one NPA (Nuclear Pitch Accent) position, which is why we can subsume them under FocusTheoryE in (22), too.

### Two general predictions

Both sub-types of FocusTheoryE in (22) entail the following two predictions:

**Uniform Marking**: There is some property that holds equally of *all* foci in the language, regardless of size, category, or grammatical function.

**Downward Syncretism**: Any broad (i.e. multi-word) focus is syncretic to one or more smaller foci.

It is probably obvious why Uniform Marking follows from FocusTheoryE in (22): any focus contains the nuclear pitch accent. As for Downward Syncretism, it should be transparent how it follows under theories on which focus “projects” from an accented terminal to dominating nodes. More generally ‘having default prosody’ is preserved under syntactic dominance: if a constituent X has default prosody, then every sub-constituent of X also has default prosody, and if X contains the NPA and is not terminal, one of its sub-constituents contains the NPA, *and* has default prosody, i.e. qualifies as a focus by FocusTheoryE in (22).

From Uniform Marking and Downward Syncretism combined, a third prediction follows, namely that each broader focus will be syncretic with *exactly one* one-word focus (since no two sub-constituents of any focus constituent can bear the NPA at the same time).

Uniform Marking and Downward Syncretism appear accurate for the Germanic languages and other languages (such as Slavic ones) for which such approaches have been developed. But they are not correct for the MorFoc languages analyzed here, as we will now discuss.

Starting with Uniform Marking, the pertinent property shared by all foci, at least in the great majority of MorFoc languages, would seem to be that they contain a focus marking morpheme, where by “contain” we mean that the focus marking morpheme is either attached to them (in the case of narrow foci) or contained in them (broader foci). This, for example, reasonably accurately describes the systems of Gùrùntùm (s.a.) or Aymara (to be discussed in Sect. 5).^17^

But there is a systematic class of counterexamples to the prediction that all foci contain a focus marking morpheme, namely *disjunctive focus syncretisms*.

#### Disjunctive focus syncretisms

In our discussion of Gùrùntùm we already saw one case of what we call, descriptively, a disjunctive focus syncretism. Recall that a sentence containing a VP of the form  is syncretic, as it can express V, Obj and VP focus.^18^

(23)
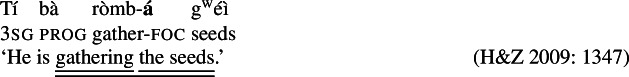

Generally, the hallmark of a disjunctive syncretism is that the same form may express focus on either constituent A or constituent B, where A and B are disjoint from one another; in the case of Gùrùntùm, either V or Obj may be the focus when the marker occurs between them.

The syncretism of V and Obj focus directly contradicts Uniform Marking: While *one* of V and Obj contains the focus marking morpheme , the other one clearly doesn’t (and this holds independently of which of the two is taken to actually contain the focus marking morpheme).

This form of syncretism is not familiar from European languages: foci on two disjoint constituents (e.g. verb vs. direct object) are never realized by the same form.^19^ They are, however, fairly common in the languages of the world. For example, it has repeatedly been observed for various languages that focus marking morphemes tend to attach to immediate constituents of the clause. Different foci, say, *within* an object are marked identically, as in the following examples from Buli and Imbabura Quechua.^20^

(24)
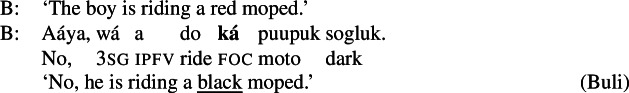

(25)
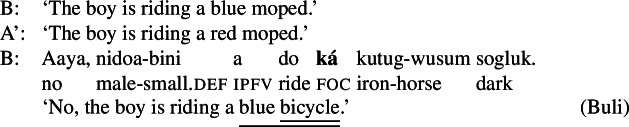

(26)
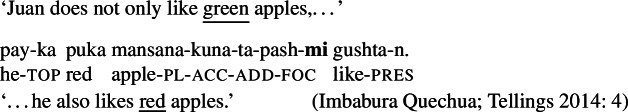

(27)
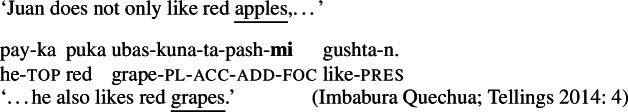

In both cases, the same focal marking may realize focus on the entire complex DP, or, disjunctively, on any of its subparts. Note that in Buli the focus marking morpheme occurs to the left of the DP, even if the focus is post-nominal, whereas in Quechua it appears at the right edge of DP, even if the focus is pre-nominal. So while in Gùrùntùm one might at first suspect that the placement of the focus marking morpheme *between* the two parts of the VP masks a syntactic ambiguity (whereby it is either attached to the left or to the right), no parallel ambiguity analysis would seem motivated for the Buli and Quechua cases.

Such cases, then, show that not *every* narrow focus contains a focus marking morpheme, and therefore directly contradict the Uniform Marking prediction.

#### Exocentric focus

We now turn to the second prediction of standard theory regarding Downward Syncretism. A glaring counter-example to Downward Syncretism is found in what we will call exocentric focus, illustrated by clausal focus in Gùrùntùm in (28) (=(5) above).

(28)
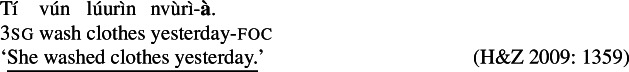

An exocentric focal marking marks a *complex* constituent (the clause in ((28))), but cannot be used for focally marking any of its subconstituents (Sbj or VP in ((28))); put differently, an exocentric focus is not syncretic to any one-word focus. We found exocentric foci in Gùrùntùm, Ewe (where a complete lack of prosodic and morphological marking can only indicate clausal focus, see Fiedler and Jannedy 2013), and Wolof (with the marker , see Sect. 3.3). It should be obvious that existing theories are ill-equipped to handle exocentric focal marking: depending on your favorite way of thinking about them, they either have potential foci go “down” to the word that bears the NPA (analogously: the focus marking morpheme), or have it project “up” from a pitch accent (focus marking morpheme) on a word; in either case, the Downward Syncretism prediction follows, which is falsified by exocentric foci.

#### The common cause, and the solution

We submit that the problem, in both cases, lies with the assumption in FocusTheoryE in (22) that the “original” focal marking would need to be on a word (or preterminal). Once we allow a focal marking to *directly* focally mark a complex constituent, as our proposal does, both disjunctive syncretisms and exocentric foci are analyzed straightforwardly. (29a) and (29b) show this for exocentric clausal focus and disjunctive VP/V/Obj focus in Gùrùntùm, respectively.

(29)
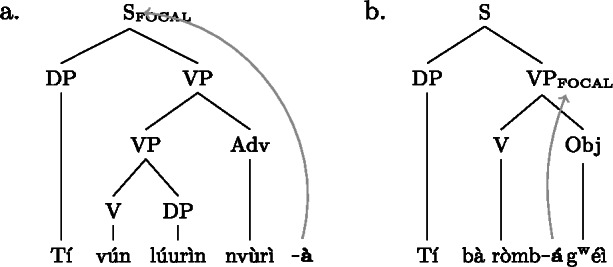

Note that now in (29a) and (29b) all *focally marked* nodes (but not all foci realized by them) do have a common property: they contain the focus marking morpheme.

Now, why does (29a) result in an exocentric focus, while (29b) results in a disjunctive focus? The short answer is: Blocking. Gùrùntùm has focal markings for Sbj and VP, which block (29a) from realizing Sbj and VP focus; but it doesn’t have focal markings for V and Obj, which is why (29b) has to be used when realizing any focus on a node dominated by the focally marked one. We will discuss this in detail in Sect. 4.4 below.

For completeness’ sake, ((30)) gives the representations for the DP internal disjunctive foci in Buli and Imbabura Quechua (cf. (24)–(27) above).

(30)
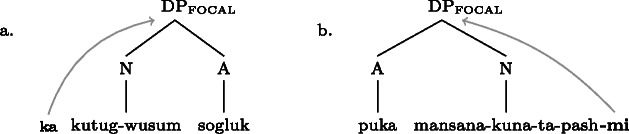

Conceivably the focal markings assumed in our analyses could translate directly into *syntactic* attachment, that is, the arrows in (29) and (30) are in fact branches of the phrase markers. This is attractive in that it helps to address the questions how focally marking is to be implemented and what the relation between the focus marking morpheme and the focally marking it expresses is; but evidently this requires a substantial amount of morphology–syntax mismatch (e.g. in (29b)), for which we lack independent evidence; we will therefore stay agnostic regarding this question.

It should be pointed out that an analysis of disjunctive focus syncretism along the lines of (29)/(30) directly makes additional predictions, owing to the fact that the apparently disjoint foci are taken to be just subparts of one encompassing focally marked constituent:

(31) If disjoint constituents A and B may be marked as narrow foci by the same focus marking …
  - a broad focus composed of A and B will be marked in the same way;
  - if the smallest constituent containing A and B contains another (disjoint) constituent C, a broad focus consisting of A+C, B+C or A+B+C will also be marked in the same way.

To illustrate, ((31a)) excludes a hypothetical variant of Gùrùntùm in which  could mark either verb focus or object focus, but not VP focus. Given that Gùrùntùm also does not distinguish different object internal foci, say A and N focus, ((31b)) predicts that in a structure , either of V+A, V+N and V+A+N can be focal (in addition to V and A+N of course); there couldn’t be a marking on which either V or A can be focal, but not V+A+N. All the languages in our sample for which we have such data confirm these predictions.^21^

This concludes our discussion of Uniform Marking and Downward Syncretism. We have argued that the most conservative extension of English-type focus theories is to give up the assumption that focal markings necessarily involve marking words, rather than phrases. In the context of focus projection theories (such as Selkirk 1984, 1995; Rochemont 1986; Schwarzschild 1999) this amounts to introducing Basic Focus Rule(s) that mark complex constituents; for other kinds of approaches this would be trickier, but could be done by *syntactically* attaching the focus marking morpheme to complex constituents.

### Defaults, syncretisms and “strange projections”

Let us now turn to the question if and how the “default prosody” part of FocusTheoryE in (22) could be adapted to the case of MorFoc languages. In looking for a default prosody equivalent, let us contemplate its *function* in the overall focus marking system of English: the default will be crucial to decide which of several sub-constituents of a broad focus will bear the NPA. For example, VP, rather than Sbj, will contain the NPA in English clausal focus (even though in either case the NPA would be within the focus) because *by default* the VP is metrically stronger than the Sbj. And within the VP, the object is by default metrically stronger than the verb, etc. (cf. e.g., Ladd 2008).

A different but equivalent way of saying this is that defaults in English are essential for determining which narrower focus a broad focus is syncretic with. This characterization holds, too, if the defaults are not taken to be purely metrical (such as “right is stronger than left”), but instead cast in terms of dedicated focus projection rules (“[F] projects from complement to head, but not *vice versa*”)—though in that case, the defaults are stipulated for the sake of correctly predicting the focus projection facts, rather than derived from general metrical properties of the language. In projection parleance, the defaults determine which nodes may “project” focus, and which may not.

Turning to MorFoc languages, two observations are crucial in this context: first, regarding their patterns of syncretisms, MorFoc languages differ greatly, not just from English, but also from one another. Second, unlike in English, where default strength in the sense relevant here is arguably correlated one-to-one with default metrical strength, MorFoc languages show no such correlates.

Elaborating on the first observation, recall, for example, that in Buli, clausal focus and *subject* focus are syncretic (“subject focus projects”) as (32) (echoing our earlier (6a)).

(32)
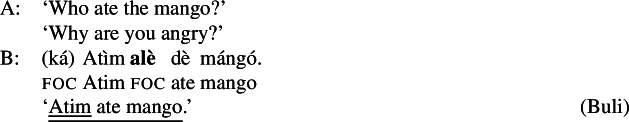

Similarly, we already saw that, depending on the language, VP focus may be syncretic to V focus, Obj focus, or to both. The full range of syncretism patterns discussed up to this point is summarized in Table 1.

**Table 1:**
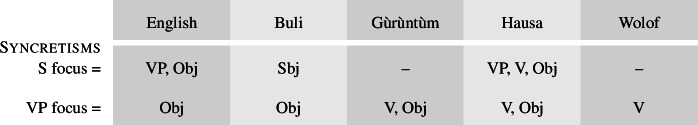
Patterns of focus syncretisms differ widely

It thus seems clear that the question of “Who gets to project?” cannot be answered universally by something like “The right sister,” “The complement,” or “The branching sister.” Nor, we think, can its answer be derived from other properties of the language in all cases, which brings us to the second observation from above.

In English, at least on the metrical view of defaults, defaults manifest independently of focus marking (i.e. the NPA): even in the background of a subject focus, it is well motivated to say that the object is stronger than the verb, as it still bears more *stress* than the verb, which speakers can hear, and instruments can measure; likewise, in a complex subject preceding a VP focus, the head noun notably (and measurably) bears more stress than a prenominal adjective. So it makes sense to say that the object bears the NPA in VP focus *because* it is “stronger by default” than the verb, because it *is* demonstrably stronger than the verb, even when it *doesn’t* bear the NPA.

In the case of MorFoc languages no such independent correlates of “strength” have been reported, and where researchers have looked for them explicitly, they haven’t succeeded (see e.g. Hartmann and Zimmermann 2007c: Sect. 5; or Rialland and Robert 2001: Sect. 2). Put plainly, an object in, say, a Buli subject focus sentence does not bear an additional focus marking morpheme , as a marker of its “strength” inside VP, nor any other property distinguishing it and other “strong” elements from their “weak” sisters.

For these reasons our analyses of MorFoc languages did not include a counterpart to “default strength” (*pace* Büring 2010). Instead, we coded the syncretism patterns directly when determining which of the constituents containing it a focus marking morpheme is taken to focally mark: Buli  is analyzed as a *clausal* focal marking—rather than a subject focal marking which for some reason can “project” to S—whereas the relative form in Hausa indeed focally marks just the subject (and hence does not “project”). Similarly, we analyzed  in Wolof as VP focal marking (since it “projects”), but  in Buli as V focal marking (since it doesn’t).

Perhaps future research will find independent properties that distinguish focal markings that project—or the phrases that host the focus marking morphemes used in them—from those that don’t, parallel to metrical strength in English; this would enable us to derive, rather than stipulate, when a focal marking goes “high” and when it doesn’t.

Until then, and given that we assume for independent reasons that complex constituents can be focally marked directly (i.e. without the mediation of “projection rules”), it seems both more parsimonious and more transparent to employ that same property of the system to analyze sycretism patterns/“projection,” without invoking defaults or “strength.”

### Oversize foci

Our analysis, in particular its account of various unusual forms of syncretisms discussed in Sects. 4.2 and 4.3, relies on the possibility of “oversize foci.” By that we mean that in a context in which, say, a narrow V focus needs to be expressed, it is instead the VP that is focally marked. Our final point of comparison with existing theories regards this feature of our proposal, and the use of Blocking to constrain it. As there is no mention of anything like Blocking in FocusTheoryE in (22), one might get the impression that this is where our proposal adds a genuine complication. But this is not the case: every complete theory of focusing will involve something comparable, as we shall discuss now.

We start by pointing out that oversized foci are technically possible in any version of alternative semantics we are aware of, all of which support the following lemma: the alternatives assigned to , as well to , are a subset of the alternatives assigned to . In words: If [F]-marking a constituent B yields the contextually required focus alternatives, [F]-marking any bigger constituent A that includes B will do the job as well.^22^ The same is true for focally marked nodes in the present proposal, as long as we ignore Blocking.

Yet it is also well-known that there must be limits to this. Otherwise, it is predicted that a focally marked VP could always be used in narrow-V or Obj focus contexts; and a focally marked clause should be usable in *any* context whatsoever. But this is of course wrong. Consider for example (33): the VP focus structure in (33a) leads to an NPA on the object, which is completely unacceptable in this context; only the structure in (33b) should be predicted to be acceptable.

(33)
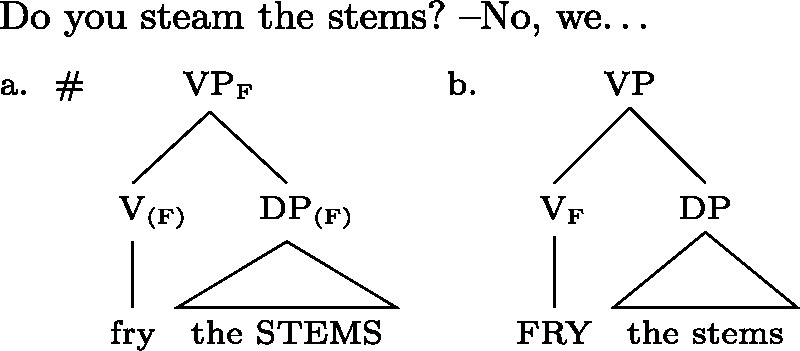

But on regular alternative semantics theories, by the lemma just mentioned, there is no alternative that (33b) has, but (33a) doesn’t. So what rules out (33a)?

The standard solution is to block oversized foci by some kind of “shrink-to-fit” clause that will enforce the use of a “smaller” focus if (pragmatically) possible. For example, Schwarzchild’s (1999) AvoidF principle will rule out (33a) in the context of the question in (33), because (33b), too, allows the alternative required in the context (that we steam the stems), while using fewer [F]-markers (for Schwarzschild, following Selkirk 1995, a VP focus with accent on the Obj requires the parenthesized [F]s on V and the Obj in (33a), or at least the latter).^23^

We call AvoidF and its kin “shrink-to-fit” clauses, because in effect they will always force focal marking on exactly the (pragmatic) focus, rather than some bigger (‘oversize’) constituent *containing* the focus. In our proposal, Blocking plays a role analogous to AvoidF; however, as stated at the outset of this section, it will not always yield a “shrink-to-fit.” For example, for Gùrùntùm, we proposed that in a case parallel to (33), (23) (repeated from (2)), the VP, rather than V, is focally marked, as shown in ((34)).

(34)
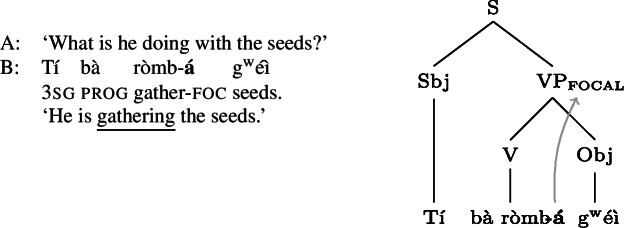

Recall that this was crucial in accounting for the fact that the same focal marking is used for VP and Obj focus as well (disjunctive syncretism). The “oversize” focus is possible, we argued, because Gùrùntùm does not *have* a focal marking for narrow V focus, so VP in (34) is indeed the smallest constituent that allows V alternatives *for which there is a focal marking*. While the focally marked constituent is still bigger than the focus, Blocking did make the focal marking “shrink-to-the-closest” (which is why clausal focal marking couldn’t be used here).

By the same token, a focally marked complex DP consisting of A and N will not compete with a narrow N or a narrow A focus (recall the discussion of examples (25)–(27) in Sect. 4.2.1) *unless the language has a distinct way of marking those*. Put generally, we predict disjunctive focus syncretisms whenever among two (or more) sister nodes, neither has a dedicated focal marking.^24^

This logic is perhaps more easily appreciated by looking at the schemata in (35); each of them represents the focal marking system of a whole language, by overlaying their individual focal markings (compare (9) and (4)). For each colored node there is a distinct focal marking (using the focus marking morpheme of the same color). Each such focal marking may realize focus on the node so colored, or any node dominated by it, *down to the next colored node*. (Again, color figures are available in online version of paper.)

(35)
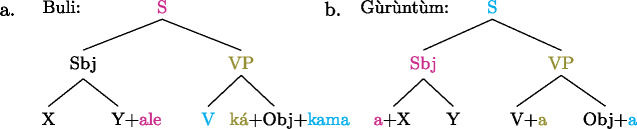

Buli  (magenta) can thereby mark focus on S (all new) or on the Sbj, or any part X or Y thereof; but it can*not* mark a VP focus, for which there is a special focal marking (olive color). Focally marking the clause in Gùrùntùm, on the other hand, (blue) cannot be used for Sbj focus, since in those one must use the more specific Sbj focus marker (magenta); nor can it be used for VP focus (or any part thereof), for which there is another specialized marking (olive). Focally marking VP in Gùrùntùm, as discussed at length in Sect. 4.2.1 may mark VP, V or object focus, as there are “focally markable” nodes within VP; in Buli, on the other hand, focally marking VP is restricted to VP or object focus, whereas V focus must be expressed by the yet more specialized V focus marker (blue).

Turning to English, we now show that the apparent “shrink-to-fit” (English) versus “shrink-to-closest” (Buli etc.) distinction is in fact an epiphenomenon. In a nutshell, there is no reason to assume that *your DOG ate my lunch* should have two different structures, depending on whether it answers *Did you eat your lunch?* or *Did my cat eat your lunch?*—What is going on, we claim, is that in the latter context (i.e. narrow N focus), since English has no way to marks a narrow N focus, it marks subject focus instead: shrink-to-closest! To spell out the argument, we invite the reader to look at English afresh, from the perspective of the present proposal. From that viewpoint, we say that at every branching node, English has the option to focally mark a daughter by making it metrically strong *when, by default, it would be weak* (see Sect. 6.4 for details, and Calhoun 2010 for a similar perspective). For present purposes we can assume that the default in English is always *w*(eak)–*s*(trong), i.e. by default the rightmost daughter of any node is metrically strong, and the other(s) weak, as in ((36a)) (but see e.g. Williams 1997: 602f for a more detailed discussion of the pertinent prosodic defaults). (36b), the English counter-part to (35) above, shows how prosodically reversing nodes—i.e. making the default-strong sister weak and prosodically promoting the default-weak sister to strong—focally marks the promoted sister (as in (35) the focally marked nodes and their focal marking, the metrically “strengthened” branch, marked by “s w” are co-colored).

(36)
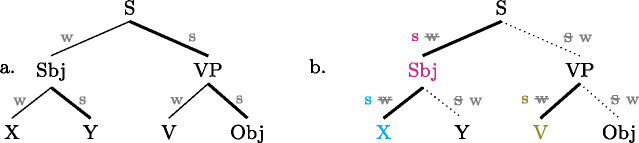

As can be seen in (36b), the result is that English has distinct focal markings for Sbj, V, pre-nominal elements X in DP, and, in general, any constituent that would, by prosodic default, be a metrically weak sister, compare ((36a)). So whenever the focus is a default-weak element in English, we do indeed get focal marking exactly for the focus (“shrink-to-fit”). Where it is default-strong, however, there is no way of marking it as focal (we cannot make it “stronger than strong”); we actually get an oversize focus, no shrink-to-fit, just like in Buli and similar MorFoc languages. For example, focus on Y in a structure like that in (36b) will be realized by prosodically strengthening the entire Sbj (relative to the VP); put differently, Sbj focus and focus on the rightmost element *within* the Sbj are syncretic. The same holds for S, VP and Obj focus: they are marked in the same way (by default stress, or, if you will, focally marking the clause; see Sect. 6.4).

The reason English oversize foci may have eluded the reader in the past is that a shrink-to-fit principle like AvoidF will force additional formal distinctions between these syncretic foci (narrow Obj versus VP versus S′ etc.) in terms of different [F]-markings, *which, however, have no effect on the prosodic realization* (whence the syncretism). We know of no empirical reasons to assume that they are in fact grammatically distinguished in the same way, say, a narrow V focus and a transitive VP focus are (see the discussion in Büring 2015). So in fact, English, just like MorFoc languages, shows the “shrink-to-closest” signature that Blocking predicts. Of course—because English uses metrical relations, rather than focus marking morphemes, for focal marking—there are more occasions on which the pragmatic focus itself can be focally marked in English than in MorFoc languages, but in many other cases, shrink-to-fit in English is simply an illusion caused by marking a distinction in the [F]-marking that has no corresponding distinction in the actual realization.

We can also explain now why English has neither exocentric foci nor disjunctive syncretisms (i.e. why Uniform Marking and Downward Syncretism hold for English), even if analyzed entirely parallel to MorFoc languages: as discussed in Sect. 4.2, these patterns emerge when among the daughters of a focally marked node there is either a dedicated focal marking for *each* (exocentric, such as clausal focal marking in Gùrùntùm, which isn’t syncretic with anyhing), or for *none* (disjunctive, such as VP focal marking in Gùrùntùm, which is syncretic with V and Obj focus), see again (35). For an English focal marking to be exocentric, all daughters would need to be default-weak (so that any of them could be specifically focally marked by making it strong); for a focal marking to be disjunctive, on the other hand, all daughters would have to be default-strong (so that none of them could be focally marked by making it strong). But metrical strength being an inherently relational concept, neither of those cases can exist. Casually speaking, a system based on the metrical weak/strong distinction, like English, cannot help but having a designated focal marking for at least one, but not all, daughter(s) of any branching node.

## Two final case studies

In this section we further illustrate our approach by way of applying it to two more languages which represent syncretism patterns not thus far discussed.

### Aymara

Aymara, an Aymaran language, spoken by about 2–3 million people around Lake Titicaca (Klose 2015), displays a syncretism between V, VP and S. This is different from Wolof-type languages discussed in Sects. 3.3 and 4.3, in which focus is only syncretic between V and VP, but not S.^25^

Focal marking in Aymara is indicated by the evidential marker  (sometimes realized as ), which in all cases appears to focally mark the constituent to its left.^26^ Accordingly, since Aymara is SOV, clausal, V and VP focus in declarative sentences are all realized by post-verbal/sentence-final . According to Hardman et al. (1988) Aymara sentences are always marked for evidentiality, and thus, also focus.^27^ Sentence, verb and VP focus are illustrated in ((37)), ((38)) and (39) respectively.

(37)
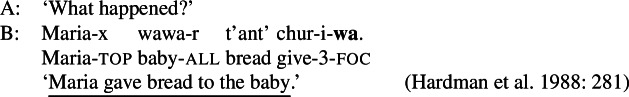

(38)
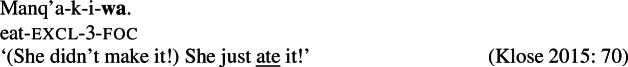

(39)
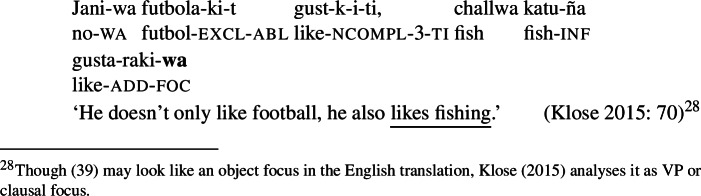

In all three examples  appears sentence-finally after the verb. Subject, object and indirect object focus are marked by  attaching at the right edge of each constituent respectively, thus creating no syncretism.^28^ Furthermore, like the focal markings of other languages discussed in this paper,  can only appear once per clause (Coler 2014). (40) summarizes these patterns.

(40)
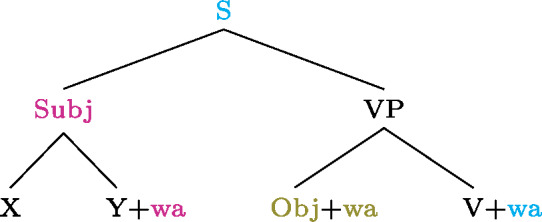

### Awing

The final type of language to be discussed here displays the limiting case of focal marking pattern, namely one where *no* two foci need to be formally distinguished. That is, anything can be the focus when there is no marking whatsoever—neither prosodically nor morphologically (languages of this type usually have ways of optional focal marking; see Sect. 7 for some examples). This is found in Awing, a Grassfields Bantu (Niger-Congo) language spoken in the Northwest Region of Cameroon by 20,000 speakers (Fominyam and Šimík 2017), but also in Ngamo (Grubić 2015), Akan (Kwa, Niger-Congo) and Ga (Kwa, Niger-Congo) (Grubić et al. 2019). Though these languages do have morphemes involved in focal marking, focus is often unmarked when it is not contrastive or exhaustive, i.e., when it is an answer to a constituent question rather than a correction. An Awing example is given in ((41)), where the sentence in B can answer any of the questions asked.

(41)
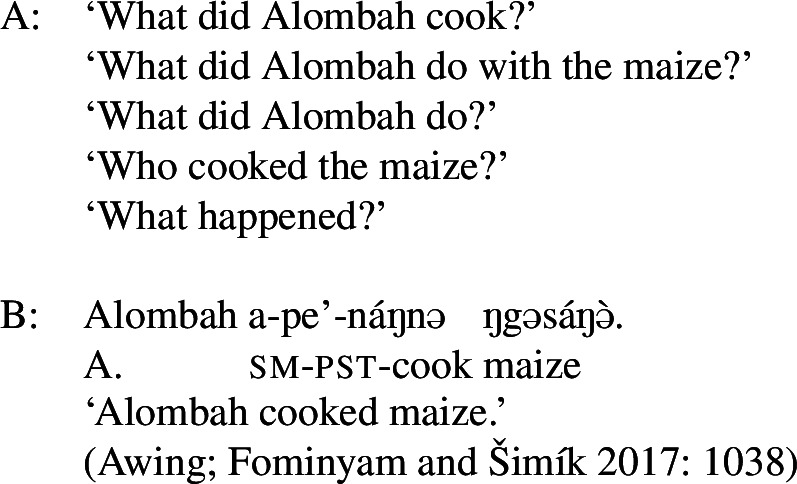

The answer in B shows that a sentence with no focal marking can be used to mark any focus in Awing: Obj, VP, Sbj and Clause. Note that even the unmarked subject can be the focus in these languages, which thus differ from Hausa (Sect. 3.2), Tangale and T’ar Barma. Since there are no focal markings in Awing, we get what we may call “completely disjunctive clausal focus.”

The various focal marking patterns discussed so far are summarized in Table 2, where syncretic foci are marked by identical color (see Appendix B for more languages that exhibit one of these patterns).

**Table 2:**
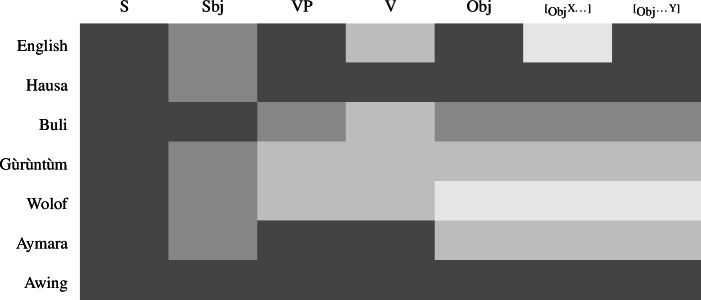
A more comprehensive table of focus syncretisms

Despite the variety, we hope to have shown that there is a common, consistent logic behind all of these systems, based on direct focal marking and Blocking. The variation can be reduced in its entirety to one factor: for which nodes in the clause does the language have a designated focal marking?

It is also worth pointing out that not everything goes: according to our analysis, syncretisms will always involve continuous sections of the tree, such as S+Sbj, S+VP, VP+V, VP+Obj etc., and combinations thereof. Technically, the sets of nodes focally marked in the same way are always such that if nodes A and B are in that set, any node dominated by A and dominating B must be, too; there could be no focal marking for, say, S and Obj, but not VP. Also, as long as we ignore optional focus movement, there is always exactly one focal marking for any given focus (i.e. no cell in Table 2 has two different colors in it).

We sidestepped, in the interest of generality, many interesting issues having to do with different kinds of focal marking systems and the related question of how the placement of the focus marking morpheme relates to the focally marked node in general. Overall, we have come across three different types of MorFoc languages: those where the same focus marking morpheme appears in different positions, like Soninke, Gùrùntùm, Aymara and Quechua; those in which different focus marking morphemes occur in the same position, like Wolof and Hausa; and those that have both different markers and different positions, like Buli and Kɔnni. Presumably, these distinctions aren’t without consequences for the way focal marking works in each language. We hope to return to these aspects in future work.

## The domain of focal markings

In this section we discuss cases of multiple focal markings within the same sentence. We argue that in the MorFoc languages, focal marking marks the maximum focus *within a clause* (which we call the domain of the focal marking), but not beyond it. Comparison with a language that has a much smaller domain of focal marking, namely English, will lead to a striking cross-linguistic prediction, which appears to be borne out.

### Multiple focal marking

All MorFoc languages we have investigated allow at most one focus marking morpheme per clause (“at most” because in some cases, a clause seems to have no focus marking morpheme at all). Though multi-clausal examples with multiple focal marking are rarely discussed in the literature (and presumably rarely used in real life), our data include some instances of this, such as (42) from Wolof. These, it turns out, give us important clues as to the precise formulation of Blocking and Focality.

(42)
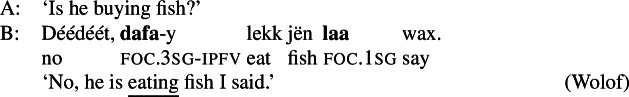

((42B)) contains two focus marking morphemes:  in the matrix clause marks the object/complement as focal, whereas  with*in* the object clause marks the embedded verb  ‘eat’ as focal.

Viewed from the perspective of the matrix clause, the  marking in the matrix makes sense: in order to correct A’s utterance, we do need the alternative ‘he is buying the fish,’ and focally marking the complement clause delivers that alternative (among many others); and, as we discussed in Sect. 4.2.2, Wolof generally does not have markers to differentiate “within-immediate-constituent” foci. Viewed from “within” the complement clause, the  marking on the embedded predicate makes sense as well, since clearly the verb, and nothing else, requires focal alternatives.

A similar example of such “multi-clausal marking,” this time from Hausa, is given in (43).^29^

(43)
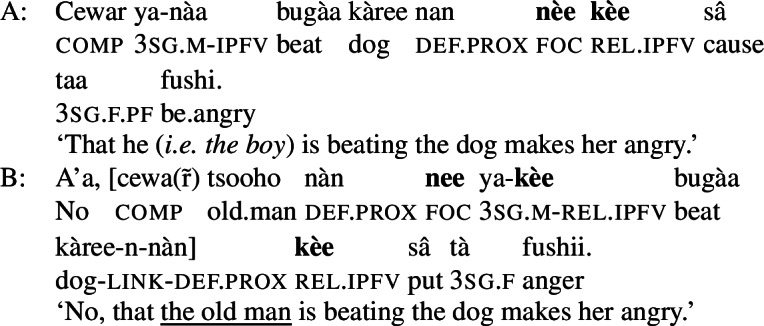

In ((43B)) the embedded and the matrix clause each show the relative form , signalling subject focus (in addition, and irrelevantly in the present context, the embedded subject is focally marked by the optional focus marking morpheme ). The entire example is a correction, and the pragmatic focus is the subject of the subject clause,  ‘the old man.’ The tree in (44) schematizes this, with the two focal nodes (and their focus marking morphemes) distinguished by different shades of blue (we use TP instead of S in the earlier trees as we need a position for the TAM markers).

(44)
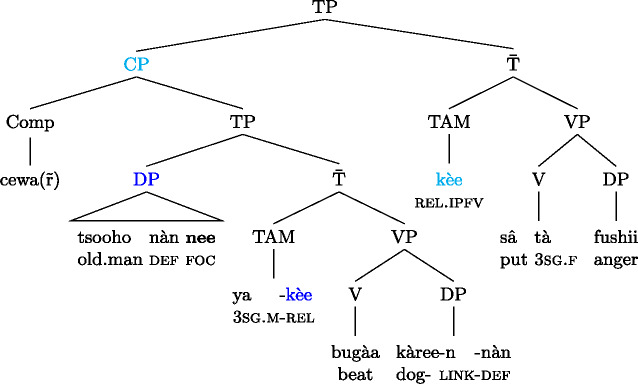

As with the Wolof example (42) above, the overall focal marking in (43)/(44) is plausible intuitively: in the matrix clause, the subject clause needs to have alternatives, and hence focal marking, and with*in* the subject, the subject ‘the old man’ needs to have alternatives; since the matrix subject is itself clausal, another, independent focal marking can be used within it.

### Blocking within a domain

The previous section aimed to show how to make sense of the double focal markings in (42) and (43) in general; yet, their precise analysis still begs theoretical questions, among them: why doesn’t the possibility of focal marking in the embedded clause block use of the broader focus marking in the matrix clause? After all, the former is more specific than the latter. This question, at least intuitively, has a simple answer. We might expect the possibility of focally marking the embedded narrow focus alone to block the use of the broader focus marking, but only when used *instead of the narrow one*. But (42) and (43) *combine* the two markings, so the structures as a whole are still as specific as can be. While, for example, the matrix  marking in (42) may be redundant (but recall that Wolof generally *must* employ *some* focus marking morpheme in each clause), B is not using a less specific focal marking instead of a more specific one; consequently, Blocking should not be invoked. This suggests the following formulation of Blocking:

(45) Blocking (official version)
  Within the domain of focal marking (in the language), use the most specific focal marking (in the language) that provides the pragmatically required alternatives.

We assume that the domain of focal marking in our MorFoc languages, including Wolof and Hausa, is the clause. Within each clause/domain, the most specific focal marking is chosen, across domains, focal markings may be combined.^30^

In languages like Wolof and Hausa, focus marking morphemes are a paradigmatic part of the verbal/aspectual morphology; from that it plausibly follows that there can be at most one focal marking per clause, and hence that the clause should be the domain of focal marking. But the “once per clause” property also seems to hold of MorFoc languages in which the focus marking morphemes are not part of the verbal/aspectual morphology, but freely placeable focus marking morphemes. As an example, consider Soninke, a Mande (Niger-Congo) language spoken by about 2,100,000 million speakers in and around Mali. Soninke has a focus marking morpheme , which immediately follows the constituent it marks; unlike in the languages discussed in Sect. 4.2.1, a possessor within DP *can* be uniquely focally marked, as in (46).^31^

(46)
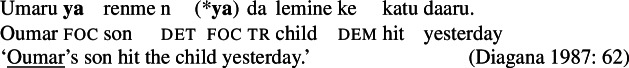

But, as also indicated in (46), there can only be a *single* focus marking morpheme on the possessor, not one on the possessor and another one on the Sbj, although putting  to the right of the subject (to express Sbj or final-element-of-subject focus) is possible in general. Semantically, additionally focally marking the Sbj as a whole should not be problematic either, for the same reason double focal marking is possible in (42) and (43). Still, evidently only one marker—and by (45) the most specific one—is possible. We conclude that the “once per clause” property is a more general property of MorFoc languages, not directly related to the morpho-syntactic nature of the individual focus marking morphemes in each language.

### Focality

Another theoretical question raised by multiple focal markings such as in (42) and (43) is: what does it mean semantically for two different nodes to be marked as focal, when one dominates the other?^32^ Take (42) again, repeated here.

(47)
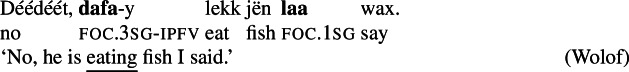

If the entire complement of  ‘say,’ is focal, what influence on the focus alternatives of the whole sentence could the embedded focal marking by  possibly have? How can (45) apply to the embedded domain if by virtue of the matrix focal marking, all “pragmatically required alternatives” are available for the sentence anyway?

Informally speaking, we want the two focal markings to “accummulate”:  says “the only constituent within the matrix to have alternatives is the complement,” and  says “the only constituent within the embedded clause to have alternatives is the verb.” Together, they should express that the only focal constituent *within the sentence* is the embedded verb. To achieve this, we need to revisit the notion of focality. In Sect. 2 we assumed that focal nodes have a rich set of alternatives (of the same semantic category), while non-focal nodes do not. We now state this more carefully.

(48) Focality (official version)
  Only focally marked nodes may vary in the alternatives of their focal marking domain.

The idea behind (48) is that alternatives to the embedded clause in (47) may only vary in the meaning of the embedded verb; they are all of the form ‘he is V-ing the fish.’ When it comes to the alternatives of the matrix clause, those may only vary in the meaning of the matrix object, informally: ‘I said S’; but the matrix object is also the domain of the embedded focal marking, so its alternatives are already restricted locally by the embedded focal marking (via (48)), effectively leaving us only with clausal alternatives ‘I said he is V-ing the fish.’ So not every focal node has the full set of alternatives, it merely *may* have them, subject to additional (accumulated) restrictions from focal markings within.^33^

### English

In the previous subsection we argued that the domain of focal marking has an important role to play in the theory of focus, and that the pertinent domain for focal marking in MorFoc languages appears to be the clause. Are there languages in which the domain of focal marking is bigger, or smaller? As we will now show, English is an example of a language with a much smaller domain of focal marking, namely the branching node. From this, a striking difference in the marking of non-constituent foci between MorFoc languages and languages like English follows, as we discuss in Sect. 6.5.

As announced in Sect. 4.4 above, we assume that prosodic reversal is the marker of focality in English; that is, reversing the metrical strength between sister nodes from the default (weak–strong in most cases) to the marked pattern (strong–weak) focally marks the newly strong node, as in (49a), as indicated by the cyan color in ((49)) (we use a dotted weak branch to remind the readers that this metrical pattern is non-default, i.e. VP in (49a) is prosodically demoted). What about the default structure? We know it can also be used in English to express certain foci, for example clausal or V(P) focus in (49b). This will follow if we assume that the *default* marks the *mother* node as focal.

(49)
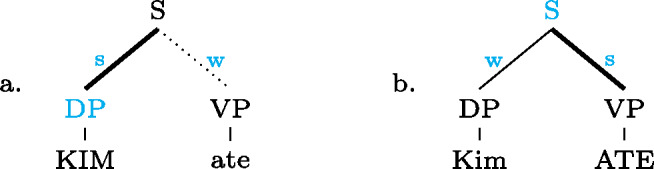

Recall that on our analysis, S in (49b) is *grammatically* marked as focal; the *pragmatic* interpretation may still be a smaller sub-constituent of S, particularly (one within) VP. Only a use as pragmatic Sbj focus, or any focus contained in the subject, is impossible, because this is blocked by the more specific focal marking in (49a). There is thus no specific structure for (49b) as V(P) focus, focally marking S is just the smallest focal marking that can be used to express VP focus; whence our use of the term focus syncretism.

The same logic applies regarding *any* branching constituent, like those in ((50)).

(50)
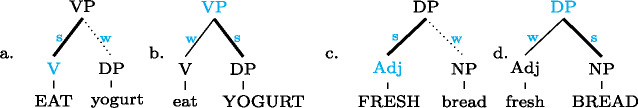

In both (50a) and (50c), stress on the default-weak, left sister focally marks that sister, while strength on the default-strong, right sister in (50b) and (50d) focally marks the mother node, which allows pragmatic focus on either the right sister alone, or the whole constituent (focus syncretism, plus Blocking.

The picture becomes more intricate once we move to trees with more than one branching constituent. Consider (51a), represented in (51b).

(51)
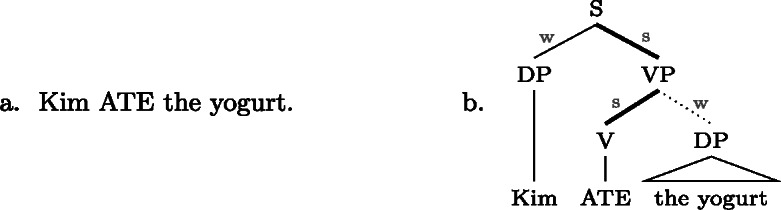

On our analysis, (52a) combines the two focal markings in (52b).

(52)
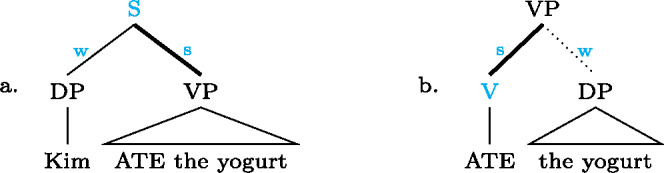

Now the domain of the focal marking comes into play again; since each branching node expresses its own focal marking, the domain of each focal marking should be *that* branching node, and nothing bigger than it. The S node in (52a) is marked as focal by the default *w*-*s* pattern among its daughters; S is at the same time the domain of that marking, so the alternatives of S may, by (48), vary in the meaning of S, or simply put: within S, any constituent(s) can be (part of the) pragmatic focus (subject of course to Blocking, which excludes (52a) from expressing Sbj or part-of-Sbj focus, because that can be expressed by the more specific *s*-*w* pattern *KIM ate the yogurt*).

Turning to the focal marking domain VP in (52b), V is marked as focal, so in VP’s alternatives, only V can vary; nothing else—*within VP*—can. This restriction on the alternatives of the focal marking domain VP again is meant to persist in the S domain; taken together, the requirements restrict possible alternatives of S to ones which, first, differ from (51a) in the verb, second, do not differ in the object, and, third, may or may not differ in the subject (note that since VP alternatives *have* to differ in V, there is no risk that Sbj could be the sole focal node in S, so Blocking by narrow Sbj focus is not an issue here). This indeed precisely characterizes the contexts (51b) is felicitous in, as shown in ((53)).

(53)
  - (Kim made the yogurt and then) Kim ATE the yogurt. (narrow V fc.)
  - (Kim ate the pickles, and then) #Kim ATE the yogurt. (#Obj focus)
  - (Kim went to the fridge and then) #Kim ATE the yogurt. (#VP fc.)
  - (The lights went out and) #Kim ATE the yogurt. (#S focus)
  - (Sam ate the yogurt before) #Kim ATE the yogurt. (#Sbj foc.)
  - (Whenever we made yogurt,) Kim ATE the yogurt. (Sbj+V focus)

Two more examples of complex (“multiple”) focal markings in English: two prosodic reversals, resulting in only one focal word, in (54a), and no prosodic reversals (i.e. “unmarked prosody”), resulting in “all focus,” (54b).

(54)
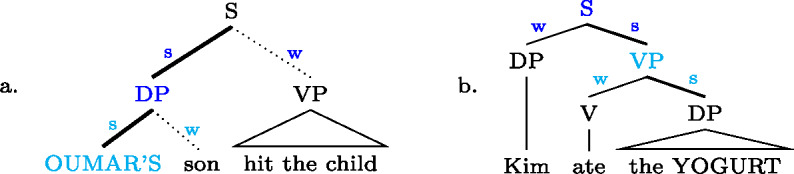

(54a) is the English counter-part to Soninke (46) above (see the tree in (55)); prosodic reversals at S and DP—indicated by the colored arrows in (54a)—mark the respective co-colored daughters Sbj and possessor as focal within S, respectively DP. We can nicely see how English, where each branching node is a domain of focal marking, uses the exact double-marking, at the S node *and* within the subject DP, that is impossible in Soninke. The resulting alternatives for S only allow variation in the possessor, each domain Sbj and S restricted on its own; so in terms of the permitted focus alternatives, the outcomes of “one marking, big domain” in Soninke and “two markings, two small domains” in English are the same.

(55)
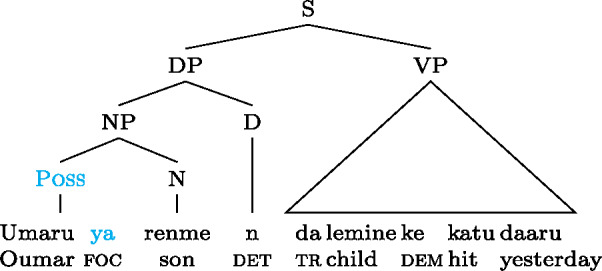

In (54b), on the other hand, all branching nodes have the default *w*–*s* pattern, “marking;’ VP and S as focal within domains VP and S, respectively. So alternatives to VP can vary in the meaning of VP, and alternatives to S can vary in the meaning of S, using all permitted alternatives to VP and Sbj—which in the case of (54b) is: all—the result being that S in (54b) has the full set of propositional alternatives; its potential for expressing focus is limited only by Blocking, given that Sbj and V (and ) could be marked specifically as narrowly focal by metric reversal. Structure (54b) is thus syncretic for object focus, VP focus and S focus, for neither of which there is a more specific focal marking.

The reader might wonder if the same results for English couldn’t have been achieved in an easier way, by claiming that *nuclear stress* (or pitch accent) directly marks the focus within the entire sentence (to be precise: marks the lowest metrically reversed node dominating the nuclear stress as the focal node within the sentence domain; this is effectively the proposal in Reinhart 2006: Ch. 3, based directly on Jackendoff 1972). Non-constituent foci like Sbj+V in (53f) crucially show that this is not sufficient: the present analysis correctly predicts that the subject in (53f) *may* be *part* of the pragmatic focus (together with the verb, which *must* be), and the object cannot be. A nuclear stress based analysis cannot predict this pattern, as neither nominal contains the nuclear stress, and no constituent includes Sbj+V but not Obj.^34^ In other words, the nuclear-stress-on-transitive-V pattern in English does not mark narrow V focus (as focal marking V does in e.g. Buli, recall Sect. 3.1), but “not-Obj” focus; this shows us that the domain of focal marking by metric reversal in English is the local branching node, not the clause or the sentence.

Two final consequences of our claim that the branching node is the domain of focal marking in English were already touched upon at the end of Sect. 4: English is predicted to have neither disjunctive syncretisms nor exocentric foci. Recall that these result if the daughters of a branching node *each* have a designated focal marking (exocentric), or if none of them does (disjunctive). But the default strong daughters in English *never* have a designated focal marking (they cannot be made metrically stronger than they already are), and default weak daughters *always* do (they can always be “promoted” by metric reversal).

### Non-constituent foci in MorFoc languages

If the conclusions from the previous subsections—that the domain of focal marking in MorFoc languages is the clause, while in English it is the branching node—are correct, this predicts that MorFoc languages should systematically mark non-constituent foci like in (53f) above differently from English. We now show that this prediction is borne out. While in English and other Germanic languages, a focus consisting of Sbj+V in a transitive clause is syncretic with narrow V focus, as just shown, the MorFoc languages for which we have Sbj+V focus data, namely Wolof, Hausa, Buli and Cuzco Quechua, invariably express Sbj+V focus the same way as clausal focus, as we now discuss case by case.^35^

In Hausa, Sbj+V focus sentences show the absolute form on the verb, i.e. look like VP, V or S focus sentences (but not like Sbj focus).

(56)
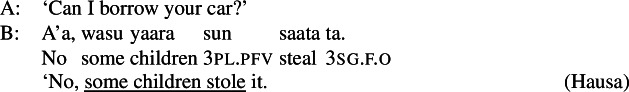

Similarly, in Wolof Sbj+V focus, as in ((57a)), is marked by the same verbal conjugation morpheme that is used in clausal focus, , in ((57b)).

(57)
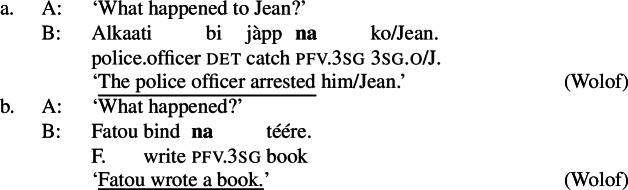

Note that this focal marking is different from that used in narrow foci in Wolof, e.g. the focus marking morpheme  focally marking the Sbj in ((58)).

(58)
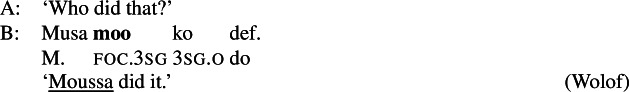

In Buli and Cuzco Quechua, Sbj+V focus is marked the same way as Sbj focus, but crucially, this is again also how clausal focus is marked.

(59)
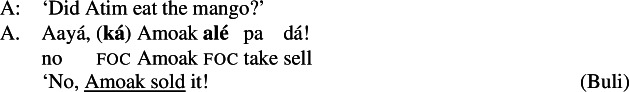

(60)
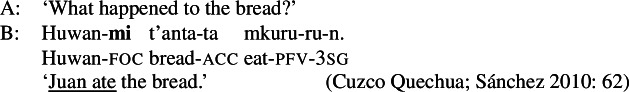

We already discussed the Buli focus marking morpheme  used for clausal or Sbj focus in Sect. 3.1 above; like in Buli, clausal focus in Cuzco Quechua is syncretic to Sbj focus (Muysken 1995), as shown in ((61)).

(61)
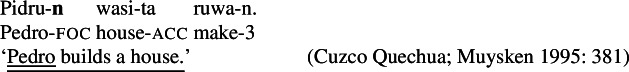

The broader generalization is again that, as in the case of disjunctive foci (cf. Sect. 4.2.1), MorFoc languages use the focal marking that focally marks the (smallest) constituent containing all parts of the focus, which in the case of Sbj+V is the clause.^36^

We close this section noting that the data just discussed are harder to make sense of from a “focus projection” perspective: why should Sbj+V focus “project” from the subject in Buli and Cuzco Quechua, from the object/VP/V in Hausa, and be exocentric in Wolof? On the other hand, if one grants that it seems logical that only clausal focal marking could encompass Sbj and V, why should this be different in English (Dutch, German…)? According to the analysis proposed here, the facts all follow from two independently established factors: the different domains of focal marking in MorFoc languages vis-à-vis prosodically marking languages like English (clause vs. branching node), and among the former, the question which focal marking ends up being the dedicated focal marking for clausal focus.

## Further considerations: Interaction with movement

In this section, we tentatively indicate ways in which focus movement interacts with Blocking and we explore potential avenues for future work. In general, there are two different kinds of focus movement: optional movement of a focused constituent, often associated with some additional pragmatic effect; and obligatory movement that is part of the language’s focus marking paradigm, the case we will start with.

### Wolof

Object focus in Wolof is always fronted.^37^ An *in situ* object cannot be interpreted as focal, whether combined with the dedicated (*ex situ*) Obj focus marking morpheme, (62b), or with the general “VP and within” focus marking morpheme, (62c) (cf. Russell 2006: 46).

(62)
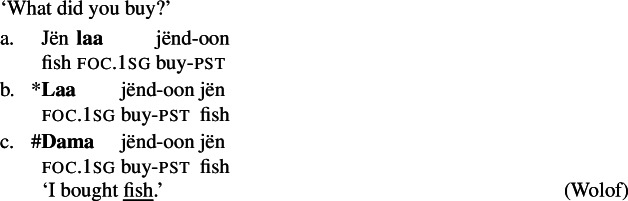

Since we know that  can mark VP focus (recall (18c) above), it must be that (62c) is ruled out by Blocking, specifically by the possibility of the *ex situ* Obj focus in (62a). In other words, the focal marking option “movement plus ” behaves the same as any other language’s Obj focus marking, *in situ* or not, within the system of Wolof.

### Hausa

Things look differently in Hausa, which allows for optional focus fronting as in (63a), as an alternative realization of the equally possible *in situ* focus in (63b) (cf. Hartmann and Zimmermann 2007c, and Sect. 3.2 above).

(63)
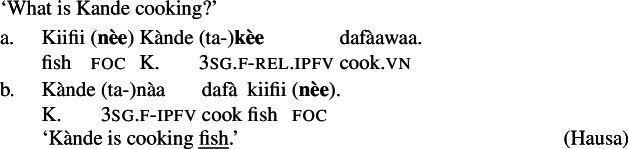

This looks as if the possibility of focus movement does *not* interact with the rest of the focus marking system at all, in particular, never blocks any *in situ* realization, even where the latter is vastly syncretic, as is the case in Hausa.

What we would like, of course, is a principled way to *predict* which focal markings “count” for Blocking (like object fronting in Wolof) and which don’t (like object fronting in Hausa); at present, we have nothing definite to offer in this regard. A promising direction could be to build on Hartmann and Zimmermann’s (2007c) observation that the fronted structures in Hausa have additional pragmatic impact, i.e. that the *in situ* and *ex situ* structures are not pragmatically equivalent (see their Sect. 3.3); assuming that the movement is motivated by such addional pragmatic effects, rather than focal marking *per se*, it would make sense that it therefore does not compete, in terms of Blocking, with the non-moved structures.

It must be kept in mind, however, that the pragmatic impact of optional focus movements is often hard to pinpoint, while on the other hand the *lack* of pragmatic impact of obligatory focus movement is *a fortiori* untestable, so this hypothesis, while attractive in principle, requires very careful study of said pragmatic effects for each language and case, which is beyond the scope of this paper.

To complicate matters further, it has been claimed for Hausa, as well as for various other languages in our sample, that subject foci string-vacuously move from the canonical subject position to a higher position in the left periphery. According to Green (2007), for example, Hausa Sbj focus sentences like (64a) have essentially the structure in (64b), with the relative form on the verb as the reflex of movement rather than—as we assumed in Sect. 3.2 above—a marker of focus.

(64)
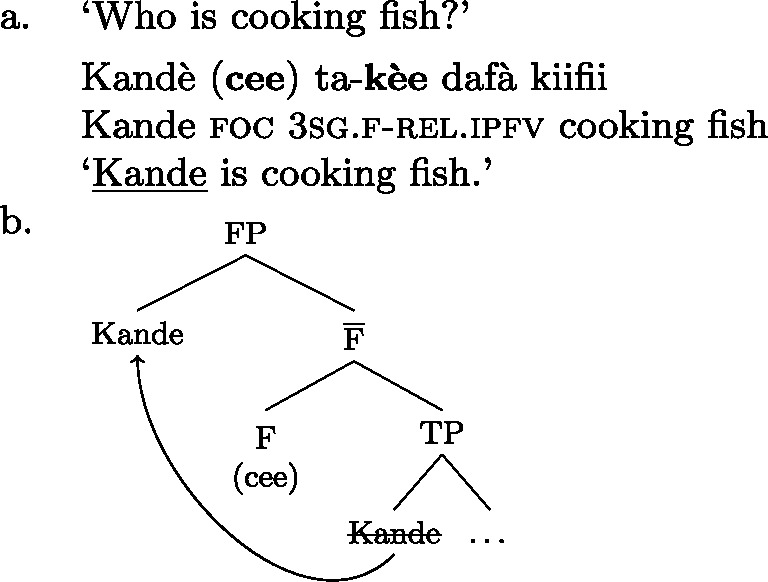

This analysis is plausible, as a comparison between (63a) and (64a) shows: In both cases, the focused constituent precedes the optional focus marker, which in both cases agrees in gender with the focused constituent, and, most importantly, in both cases the verb shows the relative form.

On such an analysis, Hausa subject focus will be completely analogous to Wolof object focus in (62). Furthermore, Sbj and fronted Obj focus in Hausa are assigned parallel structures. An unattractive consequence for the present analysis is that now even *within* Hausa, we have a split: Sbj focal marking by movement has to block *in situ* Sbj focus (resulting in obligatory movement), while Obj (or adjunct) focal marking by movement must not (whence optional movement). Hopefully, future research will unearth further regularities in this area.^38^

## Conclusion and outlook

In this paper we have laid out a theory of focus marking that applies equally to MorFoc languages and prosodically marking languages. Our aim in doing so is to connect up work on focus marking in MorFoc languages to the *theory* of focus, as developed for English and other European languages, and in particular to have the former inform the latter. And indeed, a theory able to capture both types of languages forces us, we believe, to make certain choices regarding the analysis of prosodically marking languages, in particular that complex constituents, not just words, may be directly focally marked (“no projection”), that the distribution of focus syncretisms is best understood in terms of Blocking, and that Blocking is the more generally appropriate form of something like “minimize focus.” This allows us to capture the pecularities of MorFoc languages (such as exocentric foci, disjunctive syncretisms and “strange projections”) while at the same time preserving insights of previous analyses of prosodically marking languages.

Our proposal predicts that patterns of focus syncretism are systematically restricted. That is to say, each focal marking will end up marking a “continuous” set of constituents in a tree as possible foci: the focally marked node plus, possibly, one or more nodes immediately dominated by it, plus, possibly, nodes immediately dominated by those, and so forth. The difference to “traditional focus projection” patterns is, as pointed out in Sect. 4, that syncretic markings don’t necessarily “go all the way down” to a single word; they may include more than one single word focus (disjunctive syncretism) or none at all (exocentric focal marking). Other predictions follow from the nature of the focal marking systems under investigation, particular the “once-per-clause” *versus* “once-per-branching-node” nature of the languages involved, as discussed in Sect. 6.

Semantically, we strived for maximal compatibility with Roothian alternative semantics. Our notion of “being focal” can for the most part directly be translated into “have the full alternative set of meanings of the same semantic category,” with or without mediation of syntactic [F]-markers (which, given that we do not have “projection,” serve no independent purpose); the official definition in Sect. 6.3 yields practically the same results. Blocking as used here (as well as in standard focus theories for English) remains an extraneous, competition based principle. As shown in Büring (2015), Blocking of the kind we employ *can* be implemented locally, utilizing alternative sets that include more than the literal meaning, but not *all* meanings of the same category. A demonstration of how this could be applied to the MorFoc languages analyzed in this paper has to await another occasion, however.

In many regards, our study is still exploratory. We based our proposal on data from a range of MorFoc languages, not least in the hope that even if a particular datum turns out to be different from what we know so far, the overall pattern of what does and does not occur is reasonably stable. Yet, in general, complete paradigms of various focus sizes and locations in MorFoc languages are rarely found in the literature; their elicitation is challenging for researchers and consultants alike. We expect our proposal will be much refined as more such data becomes available.

## Figures and Tables

**Table 3:**
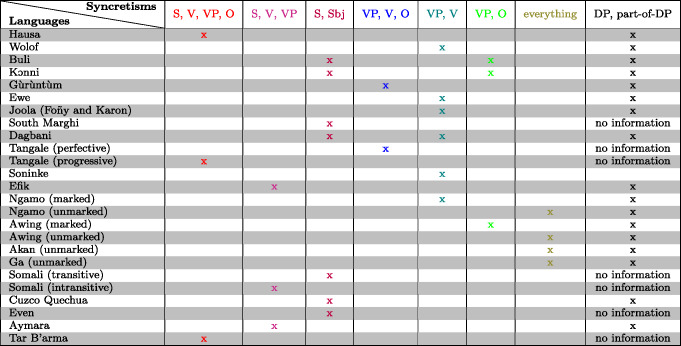
Most attested focus syncretism patterns in our sample

## Appendix A

This Appendix contains original Wolof, Hausa and Buli data we gathered in elicitation sessions (in person or by e-mail) with our consultants that were referenced in the main text and are not otherwise available.

### A.1 Sbj+O

As part of the overall patterns we were interested in double corrections. But as mentioned in footnote 37, Wolof and Hausa don’t allow for Subject+Object focus. Instead, the speakers produced bi-clausal sentences, like the Hausa example in ((65)) and the Wolof example in ((66)):

### A.2 Part-of-DP and entire DP

Following up on the discussion in Sect. 4.2.1, these data provide more examples showing that focus on modifier, noun and entire DP are focally marked the same way in Hausa and Wolof.

### A.3 Clausal focus

Following up on Footnote 37, these examples show sentences with clausal focus with subject focal marking in Wolof and Hausa—contrary to expectation. As noted, this typically involves verbs of misfortune, like those in Allerton and Cruttenden (1979).

### A.4 Sbj+Verb

The following data show that Sbj+V focus can also have subject focal marking in Wolof and Hausa. Whatever explains the fact that clausal focus can be indicated by the subject focus marking morpheme in these languages—see Appendix A.3—probably will account for this data as well.

## Appendix B: Table of focus syncretisms

See Table 3.

## Footnotes

1 See in particular Williams’s (1997) titular Blocking, or Schwardzschild’s (1999) AvoidF.

2 Moreover, the variety in syncretism patterns, as well as the aforementioned cases in which a complex focus is not syncretic to any smaller focus are equally problematic for a “default morpheme placement” idea as they are for a “universal projection” account.

3 Abbreviations: 1, 2, 3 = first, second, third person; abl = ablative; acc = accusative; add = additive; all = allative; ass = assertive; comp = complementizer; def = definite; det = determiner; dem = demonstrative; DP = determiner phrase; excl = exclamative; f = feminine; foc = focus marker; inf = infinitive; ipfv = imperfective; link = linker; m = masculine; nc = noun class; ncompl = non-completive; nmlz = nominalizer; o = object pronoun; Obj = object; pfv = perfective; pl = plural; pres = present; prog = progressive; prox = proximal; pst = past; rel = relative; S = sentence; sg = singular; Sbj = subject; sm = subject marker; T(P) = tense (phrase); top = topic; tr = transitivity marker; V = verb; vn = verbal noun; VP = verb phrase.

4 In addition,  may be placed between two phrases within a complex VP, as in (2), in which case it unambiguously expresses narrow focus on the phrase following it.

For ease of exposition, we ignore these cases of intra-VP focus in the text, but, as far as we can tell from the literature, they fit within our proposal: focus on XP within VP *must* be marked by preposing the  morpheme to that XP, blocking the more general VP marking—which would be expressed by  following the verb.

5 Analogously, (4a) actually marks that the Sbj *or something within it* is the focus; see Sect. 4.4 below.

6 A focus particle with the same form, , is found in the unrelated Mande language Jula; see Kiemtoré (2022).

7 Schwarz (2010) observes that verum is also marked with the morpheme *kámā* in Buli (and that the same holds for other Gur/Mabia languages). As an anonymous reviewer points out, this would mean that if one takes verum to be an instance of focus in Buli, it would be syncretic with verb focus. Furthermore, the morpheme *kámā* seems to be morphologically complex, consisting of the focus marking morpheme *ká*, plus *mā*.

8 Since  is optional, whenever we write  one can read it as () ….

9 Clausal/Sbj focus syncretism, exotic as it may seem from a European perspective, is quite common in African languages, as well as in other language families. Reported instances include Kɔnni (Mabia, Niger-Congo), see Schwarz (2011) and Fiedler et al. (2010); Dagbani (Mabia, Niger-Congo), see Hudu (2009); transitive clauses in Somali (East Cushitic, Afro-Asiatic), see Tosco (2002); South Marghi (Chadic, Afro-Asiatic), according to K. Hartmann (p.c.); Cuzco Quechua (IIC, Quechuan), see Sánchez (2010) and Muysken (1995); Even (Tungusic), see Matić and Wedgwood (2013).

10 VP/O syncretism is not limited to Buli. See also: Kɔnni in Schwarz (2011); Kusaal (Mabia, Niger-Congo) in Abubakari (2018); Awing (Grassfields Bantu, Niger-Congo) in Fominyam and Šimík (2017).

11 The distinction between thetic vs. categorical statements (first used in linguistic studies by Sasse 1987) have been greatly discussed in the topic/focus literature. In short, categorical statements have a bipartite structure, where one names an entity and then makes a statement about it; they are often exemplified by the topic-comment structure (see e.g. Kuroda 1972). The opposed thetic utterances do not select an entity, but express a statement as a whole; classic examples are weather sentences such as ‘it’s raining,’ and fortune/missfortune events (as classified by Sasse 1987).

12 The relative marking does not only occur in focus constructions and hence it is not only a focus marker. It also occurs in narratives, and, as the name suggests, relative clauses. Therefore, we assume that the relative form is either polysemous or ambiguous, in accordance with Newman (2000). This is the reason why, although it is used to mark a focus, we do not gloss the relative form as foc. A possible unified account of these meanings is outside of the scope of this paper (but see Zimmermann 2015 for a proposal using situation semantics), and thus the analysis we give for focus should not be extended to the other meanings of the relative form. For a full paradigm of the relative form see Newman (2000: 568) or Jaggar (2001: 153).

13 This pattern, where only subject focus has to be marked, is shared by Tangale (West Chadic) in the progressive aspect, see Hartmann and Zimmermann (2007b); and Tar B’arma (Central Sudanic, Nilo-Saharan, also known as Bagirmi), see Jacob (2010). See footnote 37 for exceptions, though.

14 Specifically, this is because the marker that indicates subject focus, such as  in (18a), consists of a subject pronoun,  in (18a), followed by the invariant morpheme . The marker that indicates non-subject focus consist of an additional  preceding the -morpheme and a subject pronoun, which in (18b) is reduced to just  instead of  for first person singular. Verb and VP focus are signaled by a marker consisting of  followed by the same -morpheme and a subject pronoun, which in (18c) is zero for third person singular. Details on how these markers can be decomposed can be found in Torrence (2013: Sect. 2). Furthermore, focus is not marked in narratives, which instead employ a so-called “narrative form,” which is unspecified for focus (Robert 1991).

15 Other languages with VP/V syncretism include: Joola Karon, see Galvagny (1984) and Sambou (2008); and Joola Foñy, see Gero and Levinsohn (1993) (both Atlantic, Niger-Congo); Ewe (Kwa, Niger-Congo), see Ameka (2010); Dagbani, see Hudu (2009); Soninke, see Diagana (1987) and Creissels (2017); Ngamo (West Chadic, Afro-Asiatic), see Grubić (2015).

16 However, in certain situations subject focus marking can also be used to convey a pragmatic clausal focus. See footnote 37 for more information.

17 There are complications, as in the case of Tangale (Hartmann and Zimmermannn 2004) and Buli, where we have different focus marking morphemes depending on the grammatical function of the constituent that is focally marked, and where in the case of focally marking V, it is not clear what local relation holds between the verb and the focus marking morpheme in a transitive clause. Perhaps such “quirks” of marking can be predicted, too, but for the time being we will not dwell on this point, which seem equally challenging for *any* approach to focus realization, the present one included.

18 This syncretism is also found in the perfective aspect in the related language Tangale (Chadic, Afro-Asiatic; see Hartmann and Zimmermann 2007b.)

19 As a reviewer points out, Swerts et al. (2002) demonstrate that in their contexts, Italian N Adj combinations (as opposed to Dutch ones) always receive accent on both the noun and the adjective, irrespective of whether the adjective is new or given. If this indeed reflects a difference in the way focus is realized in these languages, rather than a difference in focus pragmatics, disjunctive syncretisms are also attested in European languages.

20 Part-of-DP and full DP are marked identically in several languages, such as Hausa (Hartmann and Zimmermann 2007c); Wolof (see Appendix A.2); Kusaal (Abubakari 2018); Dagbani (Hudu 2009); Ngamo (Grubić 2015) and Cuzco Quechua (Sánchez 2010). For the more English-like pattern, i.e. when part-of-DP and full DP are marked differently, see the discussion of Soninke in Sect. 6.4.

21 No similar predictions are made if we literally treated disjunctive focus syncretisms as *ambiguities*. Imagine for example that the V++Obj marking in Gùrùntùm were analyzed as marking *either* the V or the Obj as focus, as indicated by the dashed lines in (32).

Nothing predicts that the focus on either one of those constituents should also be able to project; so an independent focus projection rule (FPR, solid gray line) would need to be assumed; but nothing would guarantee that the latter is part and parcel of every language that has “ambiguous focus marking.”

22 See Truckenbrodt (1995: Sect. 4.4); Schwarzschild (1999); Krifka (2001: Sect. 2); Büring (2016: Ch. 3–4) for further discussion.

23 As an anonymous reviewer points out, there are other proposals in the literature similar to the “shrink to fit” idea, cast in terms of segmental or linear closeness. See Musan (2001) for an account of this phenomenon in German, and Zimmermann (2006) for an Optimality Theory account of the expression of V/VP/OBJ-focus in Gùrùntùm and Tangale.

24 Generally, we predict syncretisms *simpliciter* exactly where there isn’t a distinct focal marking for *either* sister.

25 S/VP/V syncretism can also be found in Efik (Delta Cross, Niger Congo), see Cook (2002); it is also found in intranstive Somali clauses, where VP focus is marked the same way as clausal focus (Tosco 2002).

26 is only used in declaratives. Other suffixes are used for constituent and polar questions, but they all show the same pattern (Hardman et al. 1988).

27 There are different analyses for the -marker and there seems to be variation of its use across Aymara (M. Coler p.c.). Homola and Coler (2013) gloss it as a marker of new or non-predictable information. According to Klose (2015), the -marker is not actually a focus marker, but only associates with focus. Martinez Vera (2020), in turn, analyzes it as an amalgam of a focus particle and direct evidental. Nothing in our analysis hinges on its precise semantics, however, as already made explicit at the beginning of the paper.

28 Though  may disappear in some environments; see Klose (2015) for more details.

29 As pointed out by a reviewer, another instance in which double marking can occur in Hausa is long-distance A′-extraction, as shown in Tuller (1986) with examples such as ((43)). In ((43)) both the relative form  and the absolutive form  are grammatical on the position of the intermediate landing site of the extracted element.

The optionality of the relative form could be related to whether or not the clause is itself the focus. According to Hasiyatu Abubakari (p.c.), when a sentence such as ((42)) is embedded under a context such as A’s utterance in ((43)), only the relative form is felicitous in B’s answer.

In ((42)) both the clause ‘that they were eating food in a hurry’ and the focus ‘eating food in a hurry’ within the clause are marked. This is in contrast with long distance A′-extraction in Wolof, in which all intermediate landing sites are always obligatorily marked (Martinović 2013, 2015).

30 We deliberately coin the new term “domain of focal marking,” to be carefully distinguished from “the domain of a focus.” The latter term is variously used for either the size of the focus itself, or for the syntactic domain which consists of the focus and its background. Evidently, the domain of focal marking in our sense is different from both, extensionally and conceptually; in particular the domain of the focus in the sense of focus+background can be larger than a clause in MorFoc languages, as it arguably is in (42), and especially in (43), where the entire sentence B is contrasted with the previously utterance A.

31 Another language that does not have DP and part-of-DP syncretism when focal marking is employed is Ga (Ameka 2010).

32 Such examples correspond to what Zimmermann (2014) has termed question restriction: discourse strategies expressed by stacked (partial) questions. Thanks to an anonymous reviewer for pointing this out.

33 For a formal implementation of this, see Büring’s (2015) notion of Propagation.

34 There are various other reasons to be skeptical about a nuclear stress/accent based analysis of focus in English, including second occurrence focus and focus related shiftings of pre-nuclear stresses, see e.g. Rooth (1996); Ladd (2008); Calhoun (2010).

35 While in English it is rather easy to find all kinds of non-constituent foci such as Sbj+Obj focus, we found that in Wolof and Hausa, speakers use biclausal constructions in such cases instead (see examples in Appendix A.1). Therefore, we will only use Sbj+V data.

36 Sometimes clausal focus is marked identically to Sbj focus in Hausa and Wolof, as observed by Hartmann and Zimmermann (2007c: 18) for Hausa, and by Robert (1989: 10) and Fiedler (2013) for Wolof, and also attested in our data in Sbj+V focus sentences (see Appendix A.3, A.4). The details of when this happens are still unclear to us. Nevertheless, it seems that all these examples have an extra pragmatic import, such as unexpectedness or surprise, or that they convey misfortune and (dis)appearance (as has been observed in English in Allerton and Cruttenden 1979). The crucial difference with languages like for example, Buli, is that in Buli, clausal focus is *always* marked the same way as Sbj focus.

37 In fact, this holds for any DP focus, including, according to Martinović (2013, 2015), subject focus. We restrict ourselves to object focus for the following discussion.

38 Büring (2015) “grammaticalizes” Blocking in the form of so-called “Weak Restrictions,” which essentially mark a structural position as “not narrow focus.” This leads to an analysis for Hausa on which the *in situ* subject position, but not any other positions in the clause, is inherently marked as “not narrow focus.” The subject, when to be narrowly focused, then *must* do what other narrow foci *can* (but don’t have to) do: move to the clause initial position, which is dedicated to hosting (narrow) foci. In this way, the different patterns of (non-)optionality can be modelled; on the flip side, the general complementarity of focal marking, as embodied by the use of Blocking in the present paper, remains a meta-principle on that account, roughly that all (but not only) positions for which the language has a distinct focal marking have a Weak Restriction on them unless that focal marking is used.
